# Exposure dynamics of Ross River virus in horses – Horses as potential sentinels (a One Health approach)

**DOI:** 10.1017/S0950268824000554

**Published:** 2024-04-12

**Authors:** Nicholas K. Y. Yuen, Helle Bielefeldt-Ohmann, Mitchell P. Coyle, Joerg Henning

**Affiliations:** 1 School of Veterinary Science, Faculty of Science, The University of Queensland, Gatton, Queensland, Australia; 2 School of Chemistry and Molecular Biosciences, Faculty of Science, The University of Queensland, St Lucia, Queensland, Australia; 3 Australian Infectious Diseases Research Centre, The University of Queensland, St Lucia, Queensland, Australia; 4 Equine Unit, Office of the Director Gatton Campus, Faculty of Science, The University of Queensland, Gatton, Queensland, Australia

**Keywords:** Ross River virus, mosquito, sentinels, public health, zoonosis, meteorological data, epidemiology, One Health

## Abstract

Ross River virus (RRV), the most medically and economically important arbovirus in Australia, has been the most prevalent arbovirus infections in humans for many years. Infected humans and horses often suffer similar clinical symptoms. We conducted a prospective longitudinal study over a 3.5-year period to investigate the exposure dynamics of RRV in three foal cohorts (n = 32) born in a subtropical region of South East Queensland, Australia, between 2020 and 2022. RRV-specific seroconversion was detected in 56% (n = 18) of foals with a median time to seroconversion, after waning of maternal antibodies, of 429 days (95% CI: 294–582). The median age at seroconversion was 69 weeks (95% CI: 53–57). Seroconversion events were only detected between December and March (Southern Hemisphere summer) over the entire study period. Cox proportion hazards regression analyses revealed that seroconversions were significantly (*p* < 0.05) associated with air temperature in the month of seroconversion. Time-lags in meteorological variables were not significantly (*p* > 0.05) associated with seroconversion, except for relative humidity (*p* = 0.036 at 2-month time-lag). This is in contrast to research results of RRV infection in humans, which peaked between March and May (Autumn) and with a 0–3 month time-lag for various meteorological risk factors. Therefore, horses may be suitable sentinels for monitoring active arbovirus circulation and could be used for early arbovirus outbreak detection in human populations.

## Introduction

Understanding the exposure dynamics of zoonotic vector-borne diseases is important to inform public health measures, such as the provision and planning of mosquito control programmes. However, such studies are difficult to conduct in human populations as seroprevalence data are often derived from clinically ill patients. Thus, subclinical infection rates in humans are not known and cannot be incorporated into infectious disease models. Sentinel animals, that is, naïve animals strategically placed for the monitoring of incursion and transmission of economically and medically important exotic and endemic diseases, are therefore often used in lieu [1]. Livestock, such as sheep and cattle, are routinely used as sentinel animals for the Australian National Arbovirus Monitoring Programme [2]. However, ruminants are not relevant to the transmission of Ross River virus (RRV), as they only express a variant of the alphavirus receptor, Mxra8, which cannot bind RRV [3], and hence they are not involved in the transmission dynamics of RRV [4].

Ross River virus is an alphavirus, belonging to the family *Togaviridae*, causing most arboviral infections in Australia, with an average of around 5,000 case notifications annually [5]. RRV transmission is maintained between the reservoir host, such as marsupials (e.g., brushtail possums and wallabies), and mosquitoes [5]. Impressively, more than 40 species of mosquitoes have been identified to be capable of transmitting RRV [6, 7]. To date, only humans and horses have been confirmed as susceptible hosts of RRV disease. Infected humans are reported to experience fever, joint pain, and lethargy [8–11], with some patients experiencing chronic clinical signs of more than 12 months’ duration [8, 10, 11]. Published case studies/series have consistently reported similar clinical signs in horses to those described in infected humans [12–15].

Previous research identified high seroprevalence of RRV in horses in a subtropical region (Lockyer Valley) of South-East Queensland (SE QLD), Australia [16]. This region is characterized by intensive horticulture production and has extensive pastures that support a high density of horses. It is adjacent to large metropolitan areas, including Brisbane, the Gold Coast, and the Sunshine Coast, where millions of people reside, and large cohorts of tourists visit at all times of year. The year-round presence and interaction between marsupials (reservoir hosts), the mosquito population (vectors), and horses (susceptible hosts) in this region [16] provide a unique opportunity for the study of exposure dynamics of RRV and for conducting an assessment of the potential usefulness of horses as sentinel animals for RRV transmission.

Vector-borne disease transmission cycles are depended on reservoir host and vector dynamics, which is influenced by meteorological conditions. These factors fluctuate in different parts of Australia depending on landscape features and local weather. Various types of predictive models have been developed to identify environmental risk factors associated with increased RRV transmission. It appears that rainfall, with a lag period of 0 to 3 months, is the most frequently identified meteorological risk factor for RRV outbreaks in humans across Australia [5, 17–24]. Temperatures (both minimum and maximum) and relative humidity, with 1–2 months lags, were also frequently identified as environmental drivers for increased RRV notification in humans [18–21, 23]. Significant RRV hotspots for humans have been identified in peri-urban suburbs in SE QLD, Australia, where residential areas, agricultural practice, and conserved natural landscapes intersect [25].

The study presented here aimed to determine the exposure dynamics of RRV in horses over a 3.5-year period. Specifically, (i) estimation of the rate of waning of RRV-specific neutralizing maternal antibodies in foals, (ii) estimation of the rate of acquiring natural infection of RRV in naïve horses, and (iii) identification of meteorological variables (as a surrogate for vector activity) that are associated with RRV infection in naïve horses. This represents the first longitudinal study to identify environmental risk factors of RRV transmission in an agricultural region in Australia.

## Material and methods

### Study area

**Figure 1.**
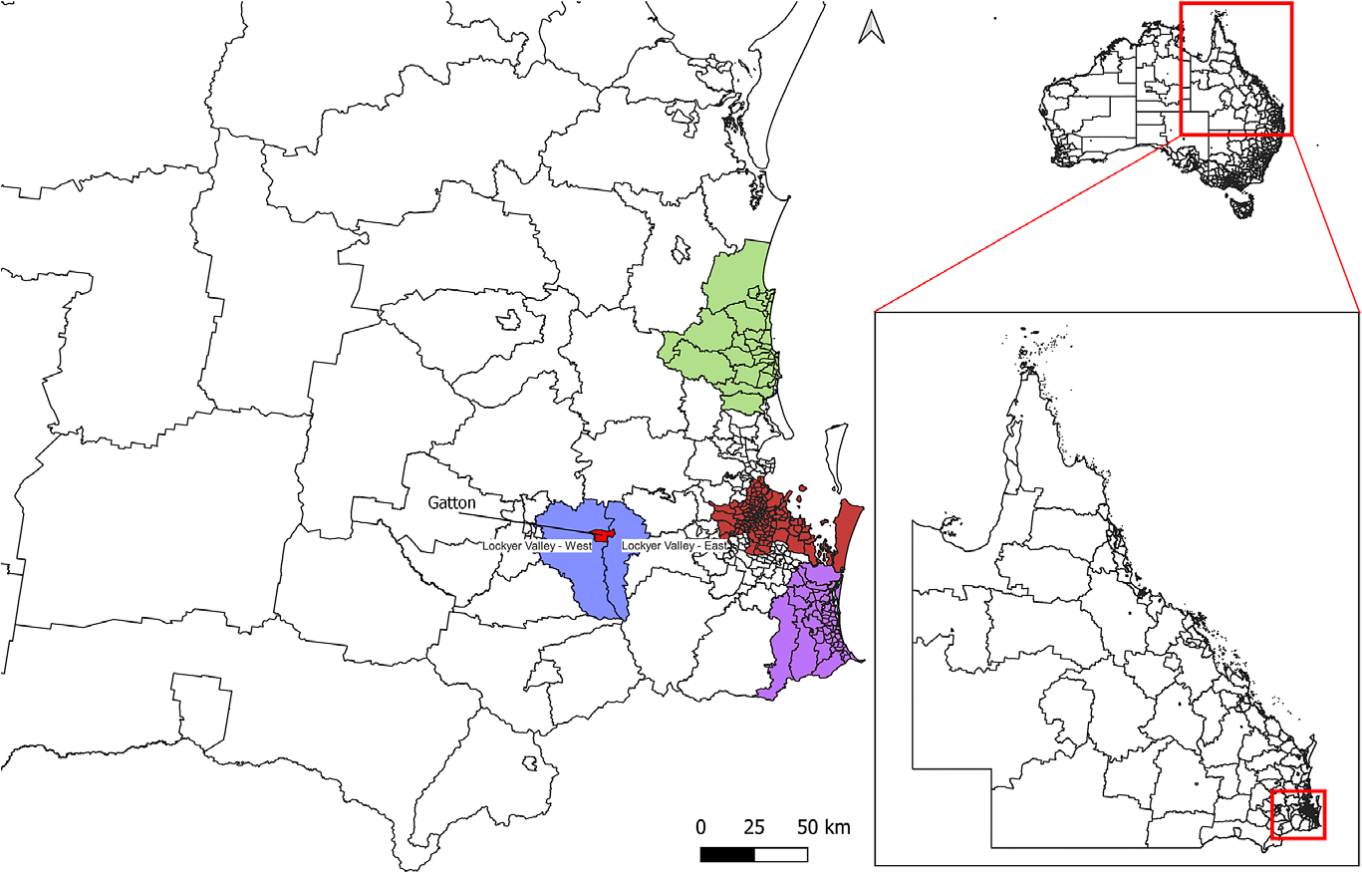
Map of South East Queensland, Australia, with boundaries of administrative areas, indicating where a longitudinal study on Ross River virus infection was conducted. The Lockyer Valley administrative area, where the study site was located, is coloured in blue, and the administrative centre of Gatton is shown in red. The study area is adjacent to three densely populated areas: Brisbane (brown) located to the east of the Lockyer Valley, the Gold Coast (purple) to the south-east, and the Sunshine Coast (green) to the north-east.

We conducted a prospective longitudinal study over a 3.5-year period, commencing 1 August 2020 and ending 31 June 2023. The study was conducted in a subtropical region of SE QLD, Australia, in the local administrative area of the Lockyer Valley (Figure 1). This region is characterized by intensive horticulture production, which include cucurbits, legumes, *Brassica* spp., corn, and sorghum. Fertile and well-irrigated soils in the region also provide excellent pastures for horses. The study area is adjacent to the administrative areas of Brisbane, the Gold Coast, and the Sunshine Cost. The rural University of Queensland (UQ) Gatton Campus is located in the heart of the study region. The campus covers an area of more than 1,000 ha, with approximately 100 horses at any one time within a year, and also incorporates crop and grazing fields, dairy and sheep production, a piggery, and a wildlife centre [26]. Marsupials such as red-necked wallabies (*Notamacropus rufogriseus*), Northern brown (*Isoodon macrourus*), and long-nosed (*Perameles nasuta*) bandicoots, common brushtail possums (*Trichosurus vulpecula*), rufous bettong (*Aepyprymnus rufescens*), and Eastern grey kangaroos (*Macropus giganteus*) are regularly reported in the study area [27].

Foals born and residing at the Equine Unit on the UQ Gatton Campus from 2020 to 2022 (n = 32) were enrolled in this study. Each cohort of horses born in the same year were agisted together in the same paddock, with regular paddocks rotations, throughout the study period. All paddocks were irrigated periodically as required depending on pasture availability and rainfall. Water troughs, with automatic filling system, were present in all paddocks and hay were supplemented as required depending on pasture quality and availability. Paddocks were free of trees with shelters provided.

### Sample collection

Blood samples from foals were collected via jugular venipuncture using a 20G hypodermic needle pre-suckle, post-suckle (24 h post foaling), then approximately monthly until six months of age, after which foals were weaned and sampled opportunistically approximately every two to three months. Blood samples from mares were collected at the time of foaling. No clinical signs were recorded from any animals seroconverted to RRV. Animal ethics approval was obtained from The University of Queensland (UQ) Production Animal Ethics Committee (permit numbers SVS/344/18 and 2021/AE000763) prior to commencement of the study.

### Cell culture and virus amplification

Cell culture and virus amplification were performed as described previously [16]. Briefly, Vero cells (African green monkey (*Chlorocebus* sp.)-derived kidney epithelial cells) and C6/36 cells (*Aedes albopictus* larvae mid-gut cells) were cultured as previously described [28]. The virus seed stock used in this study was RRV isolate Cairns 2007 [29]. C6/36 cells were used for virus stock amplification. Amplified virus stock was stored at −80 °C in 1-mL aliquots. Virus titre was determined by virus titration assay as previously described [28] and calculated as TCID_50_ infectious units/mL.

### Virus neutralization test

Virus neutralization tests (VNT) were performed as previously described [16]. Briefly, each heat-inactivated serum sample was tested for the presence of neutralizing antibodies to RRV in duplicates serially diluted from 1:20 to 1:160. Positive (no serum) and negative (no virus) controls were included in each microtitre plate.

### Meteorological data

Half hourly weather data collected from the UQ Gatton Campus weather station (station no. 040082) between 1 August 2020, and 3 July 2023, were made available for analysis. The weather station is owned and maintained by the Bureau of Meteorology (Australian Government). Due to equipment failure, no data were available between 26 February 2022, and 27 May 2022. Time periods representing the highest mosquito activities were used, i.e. dawn and dusk [30]. Air temperature, dew point temperature, relative humidity, wind speed, and wind gust speed between 5 am–8 am (dawn) and 4 pm–7 pm (dusk), and daily cumulative rainfall were extracted. The three-hourly data for dawn and dusk were averaged, respectively, to represent the morning and afternoon mosquito activity. The averaged dawn and dusk data were then further averaged to represent the daily average data. Missing data points were computed using a multiple imputation approach (see Section title “*Statistical analysis*”).

### Statistical analysis

Seropositivity was defined as a RRV neutralizing antibody titre >1:20. Depending on the temporal stage of seropositivity, each sample was assigned to group ‘0’, ‘1’, or ‘2’. Foals with RRV maternal antibodies detected after colostrum intake were noted as ‘1’. The first sample that tested negative to RRV neutralizing antibody after colostrum intake was assigned as ‘0’. Once natural infection was acquired, foals were assigned as ‘2’. Therefore, for the purpose of maternal antibody waning analysis, the failure event was detected when sample group changed from ‘1’ to ‘0’; and for natural infection, when ‘0’ changed to ‘2’.

Days for the waning of protective RRV neutralizing maternal antibodies and days from seronegative till RRV seroconversion were visualized using Kaplan–Meier survival curve and median survival time with 95% confidence intervals were calculated for these time periods [31]. Day at risks for RRV infections commenced from the date when the first RRV-seronegative sample was collected after colostrum intake. Log rank tests were performed to compare survival time in waning of maternal antibodies and time till RRV seroconversion across years [31]. The correlation between days till RRV seroconversion and days until loss of maternal antibodies was displayed in a scatter plot and quantified by the calculation of the Pearson’s correlation coefficient.

Time series analysis was performed on the meteorological daily data. Missing data were computed using multiple imputation [32]. For this, a univariate predictive mean matching imputation method with nearest neighbours set at 21 observations and posterior estimates calculated from a bootstrap sample was applied. Time series decomposition [33] was performed for each meteorological variable by year using the unobserved-component smooth-trend model with a seasonal component. Post-estimation prediction of unobserved components of trend, seasonal, and residuals were calculated using all sample information.

Bivariate Pearson’s correlation coefficients were calculated to explore the correlation between any two meteorological variables. If high correlation was identified (r > 0.95), one of the two variables was omitted.

To investigate the impact of the meteorological variables on the time till seroconversion in foals, semi-parametric Cox proportion hazards regression was used [31]. As seropositivity for RRV was determined approximately every month, daily averaged meteorological data were averaged per month and daily cumulative rainfall was summarized per month.

Three analytical approaches were used to investigate the impact of meteorological variables on time until seroconversion. In the first approach, individually monthly meteorological variables in their original units of measurements were considered as time-varying covariates with lag periods of up to two months. In the second approach, correlation between monthly meteorological variables was accounted for by developing principal components of meteorological variables and the estimated components scores were used as covariates in the analysis. In the third approach, meteorological variables were standardized by calculating the percent change of a monthly mean in comparison with monthly mean in the previous month, and the percent changes of individual covariates were used in the analysis.

The proportional hazard assumption was tested using Schoenfeld residuals and the link test [31]. Covariates with hazard ratios at *p* < 0.2 in the univariate models were considered for inclusion in the multivariate Cox regression models using a stepwise forward model building strategy. Likelihood-ratio tests were performed to compare models. Confounding was assessed by individually adding each covariate back to the final model, retaining any variables that resulted in >20% change in hazard ratio of covariates already in the model. Goodness of fit of the final model was assessed using Cox–Snell residuals. Statistical analyses were performed in Stata BE 17.0 (StataCorp. 2021. Stata Statistical Software: Release 17. College Station, TX: StataCorp LLC).

## Results

### Time for waning of RRV maternal antibodies and time until RRV natural infection

**Figure 2.**
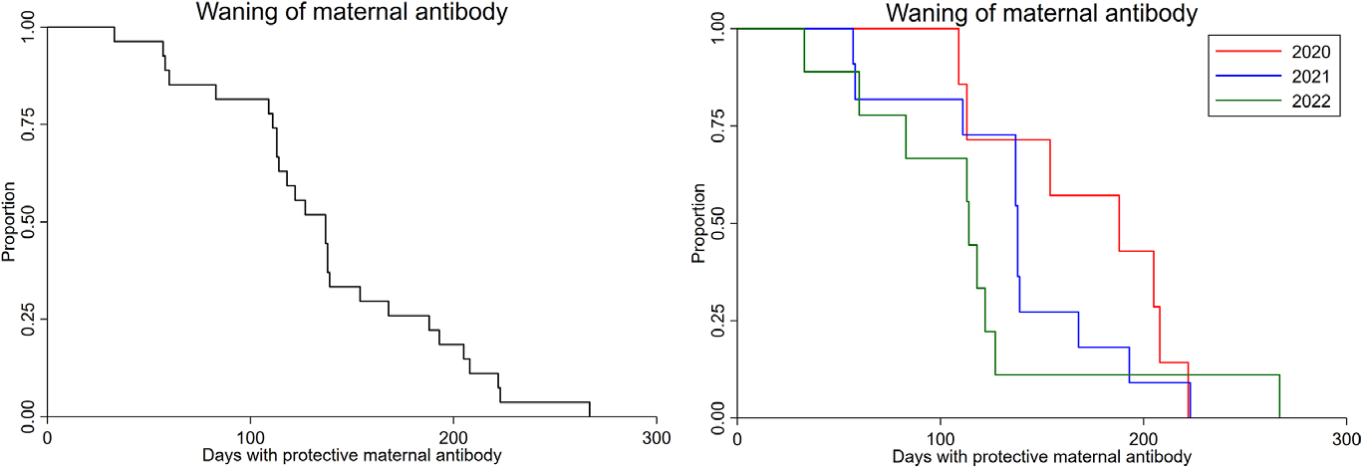
Kaplan–Meier survival curves for waning of Ross River virus maternal antibodies in foals across all birth years (2020–2022, n = 27) (A), and by each year in which foals were born (B). There was no differences in the survival times for waning of Ross River virus maternal antibodies between birth years (Log rank test *p* = 0.45).

**Figure 3.**
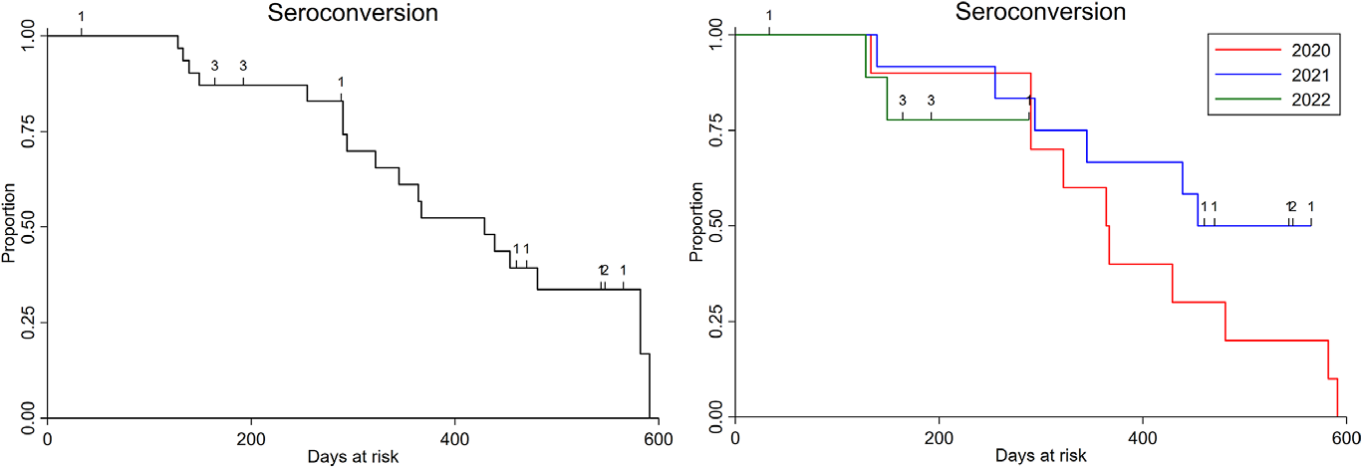
Kaplan–Meier survival curves for Ross River virus seroconversion in foals across all birth years (2020–2022, n = 32) (A), and by each year in which foals were born (B). Numbers indicate right-censored animals. There was no difference in the survival times for Ross River virus seroconversion between birth years (Log rank test *p* = 0.31).

All pre-suckle samples were negative for RRV-specific antibodies. A total of 27 foals had neutralizing antibodies against RRV after colostrum intake. Foals (n = 5) born to RRV-seronegative mares remained seronegative to RRV after colostrum intake. The overall median time for the waning of RRV-neutralizing maternal antibodies in foals (n = 27) was 137 days (95% CI: 113–154), which is equivalent to 4.6 months (Figure 2, Supplementary Table S1). The overall median time from seronegative till seroconversion was 429 days (95% CI: 294–582), with the median age at seroconversion being 69 weeks (95% CI: 53–75). The overall cumulative incidence risk of seroconversion was 56% (95% CI: 38–74) over the study period of 3.5 years, with a yearly incidence risk of 18% (95% CI: 5–40) and 36% (95% CI: 19–56) in 2021 and 2022, respectively, and 22% (95% CI: 6–48) for the 6-month observation period in 2023. All foals from the 2020 cohort (n = 10) had seroconverted to RRV by the conclusion of the study (31 June 2023); however, six and eight foals from the 2021 and 2022 cohorts, respectively, remained seronegative (right-censored) at the end of the study period (Figure 3, Supplementary Table S1). The days for maternal antibodies to wane and time until seroconversion were not correlated (r = −0.0062; *p* = 0.98; Supplementary Figure S1).

### Meteorological data

The daily averaged values for each meteorological variable were averaged by month and were presented by years in Table 1, and results are visualized in the Supplementary Material (Supplementary Figures S2–S13). Overall, air temperatures follow a seasonal pattern: spring (September to November), summer (December to February), autumn (March to May), and winter (June to August). Increased daily cumulative rainfall was observed between October 2021 and July 2022. All seroconversion events occurred between December and March each year.

**Table 1.** Monthly summary statistics of meteorological variables collected from UQ Gatton weather station between 2020 and 2023. Temperature, wind, and humidity data are presented as mean ± s.d. Rainfall data is presented as cumulative monthly rainfall (mm)

Variable	Year	Month													
		1	2	3		4		5	6	7	8	9	10	11	12
Air temperature (°C)	2020										14.2 ± 5.4	18.4 ± 5.8	21.0 ± 4.8	24.0 ± 5.3	25.8 ± 4.6
	2021	24.5 ± 3.8	25.0 ± 4.2	22.8 ± 3.7		18.1 ± 4.7		15.0 ± 4.5	12.2 ± 4.4	12.5 ± 4.5	14.3 ± 5.6	16.7 ± 5.8	21.1 ± 4.8	21.8 ± 3.3	23.9 ± 3.4
	2022	24.4 ± 3.5	23.1 ± 4.1						11.7 ± 4.3	11.7 ± 4.3	13.4 ± 5.1	16.7 ± 4.3	19.5 ± 4.2	21.1 ± 4.8	22.7 ± 4.5
	2023	24.6 ± 4.1	24.6 ± 4.8	24.1 ± 4.5		18.6 ± 4.5		13.6 ± 5.4	13.0 ± 5.2						
Dew point temperature (°C)	2020										5.1 ± 4.4	8.1 ± 5.7	12.1 ± 3.6	13.2 ± 3.5	17.6 ± 3.1
	2021	17.6 ± 1.9	17.1 ± 2.1	17.7 ± 3.1		12.0 ± 4.1		11.5 ± 4.2	8.0 ± 3.8	7.8 ± 4.3	7.9 ± 4.4	8.0 ± 4.5	13.6 ± 5.2	16.4 ± 4.7	18.1 ± 2.3
	2022	19.4 ± 2.0	17.5 ± 3.1						6.5 ± 3.2	7.3 ± 3.5	7.7 ± 4.1	11.1 ± 3.2	14.6 ± 2.9	11.8 ± 4.7	14.8 ± 3.5
	2023	17.9 ± 1.8	17.4 ± 2.4	18.4 ± 2.8		12.6 ± 3.1		6.7 ± 4.2	6.9 ± 4.7						
Wind speed (knots)	2020										6.6 ± 3.5	7.1 ± 3.9	6.6 ± 3.9	7.8 ± 4.6	7.7 ± 3.9
	2021	7.2 ± 4.1	6.9 ± 4.4	5.8 ± 3.4		6.0 ± 2.4		5.7 ± 2.8	5.3 ± 3.3	5.9 ± 4.2	6.4 ± 3.3	6.7 ± 3.5	6.3 ± 3.5	6.4 ± 3.3	6.1 ± 3.8
	2022	6.9 ± 3.6	7.1 ± 3.8						5.0 ± 3.9	5.0 ± 3.4	4.3 ± 2.6	4.2 ± 2.1	4.5 ± 2.5	6.3 ± 3.0	5.9 ± 3.4
	2023	5.5 ± 3.9	5.5 ± 3.3	3.8 ± 2.9		4.7 ± 2.7		4.6 ± 3.1	4.0 ± 3.0						
Wind gust (knots)	2020										9.4 ± 4.9	10.1 ± 5.3	9.7 ± 5.1	11.4 ± 6.0	10.9 ± 5.2
	2021	10.5 ± 5.5	9.9 ± 6.0	8.2 ± 4.5		8.2 ± 3.4		7.7 ± 3.5	7.3 ± 4.4	8.3 ± 5.7	8.7 ± 4.5	9.3 ± 4.7	9.0 ± 4.7	9.2 ± 4.5	9.2 ± 5.3
	2022	9.9 ± 5.0	10.4 ± 5.4						7.1 ± 5.2	7.4 ± 4.9	6.4 ± 3.4	6.2 ± 2.9	6.7 ± 3.4	9.3 ± 4.3	9.0 ± 4.6
	2023	8.4 ± 5.5	8.4 ± 4.7	5.8 ± 4.1		6.9 ± 3.8		6.6 ± 4.4	5.9 ± 4.1						
Relative humidity (%)	2020										59.5 ± 22.8	56.7 ± 21.7	61.4 ± 20.5	55.0 ± 17.2	64.4 ± 18.3
	2021	68.1 ± 15.5	65.5 ± 17.4	77.8 ± 18.0		71.5 ± 19.5		77.8 ± 16.6	78.0 ± 16.8	75.6 ± 18.5	69.2 ± 20.2	62.4 ± 23.1	67.6 ± 22.5	74.5 ± 18.0	72.6 ± 15.3
	2022	75.9 ± 14.7	73.8 ± 17.5						73.4 ± 16.6	77.4 ± 17.1	73.1 ± 20.1	73.3 ± 19.7	76.3 ± 17.3	59.4 ± 19.1	64.8 ± 18.8
	2023	69.2 ± 17.0	68.3 ± 18.9	75.0 ± 20.7		71.4 ± 19.0		68.0 ± 21.7	70.8 ± 21.3						
Cumulative rainfall (mm)	2020										30.8	1.8	80.4	8	68.8
	2021	86	49	143		71.6		95.4	18.6	83	5	15.6	122.2	277.4	139
	2022	91	144.2						17.2	67.8	26.2	78.4	138.6	21.6	86.6
	2023	72.8	13	74.6		20.2		55.4	6.2						

*Note:* Boxes coloured in green indicate events of seroconversion occurring in respective months; boxes in grey refers to no data available (i.e. months outside the study period or months with failure of data recording equipment).

As expected, all meteorological variables were significantly correlated. Notably, wind speed and wind gust were highly correlated (r = 0.987, *p* < 0.0001); and monthly cumulative rainfall and monthly average rainfall were highly correlated (r = 0.9707, *p* < 0.0001). As the maximum wind speed would have a greater effect on mosquito dispersion than averaged wind speed, wind gust was chosen as the variable to be retained. Cumulative monthly rainfall was considered more important as a proxy for mosquito-breeding grounds than average monthly rainfall and was retained. Therefore, five meteorological variables (monthly averaged) were considered as covariates for time until seroconversion, namely air temperature, dew point temperature, relative humidity, wind gust, and cumulative rainfall.

### Association between change in meteorological variables and time until new RRV infection

#### Modelling approach 1: Impact of individual meteorological variables in original units of measurement on seroconversion

The univariate analysis revealed that monthly air temperature, dew point temperature, and relative humidity (time-lag 0) were significantly (*p* < 0.05) associated with time till RRV seroconversion (Table 2). Every unit increase in air temperature and dew point temperature slightly increased the hazard ratio, while relative humidity slightly decreased the hazard ratio. The meteorological variables in the previous 2 months (time-lag 1 and 2) were not significantly (*p* > 0.05) associated with RRV seroconversion (except time-lag 2 for relative humidity). Wind gust and cumulative rainfall were not significantly associated with RRV seroconversion (*p* > 0.05) under modelling approach 1. Using air temperature (without time lag) as the base model, the addition of each meteorological variable did not result in a significant (*p* < 0.05) improvement of the final model, nor was any confounder identified. Therefore, the univariate regression results represent the final models for this modelling approach (Table 2). Goodness-of-fit results are shown in Supplementary Figure S14.

**Table 2.** Univariate Cox regression results of the association between time till Ross River virus seroconversion and individual meteorological covariates expressed in original units of measurement, with time-lags (modelling approach 1)

Variable	Time-lag	Hazard ratio (95% CI)	*p* value
Air temperature (°C)	0	1.05 (1.01, 1.08)	**0.013**
	1	1.01 (0.98, 1.04)	0.539
	2	0.99 (0.96, 1.01)	0.314
Dew point temperature (°C)	0	1.03 (1.00, 1.06)	**0.039**
	1	1.00 (0.98, 1.03)	0.773
	2	0.97 (0.95, 1.00)	0.058
Relative humidity (%)	0	0.98 (0.96, 1.00)	**0.042**
	1	1.00 (0.98, 1.01)	0.879
	2	0.98 (0.96, 1.00)	**0.036**
Wind gust (knots)	0	1.14 (1.00, 1.30)	0.051
	1	0.97 (0.90, 1.04)	0.406
	2	0.98 (0.92, 1.05)	0.582
Cumulative Rainfall (mm)	0	1.00 (1.00, 1.00)	0.566
	1	1.00 (1.00, 1.00)	0.704
	2	1.00 (1.00, 1.00)	0.231

Bold entries highlight *p*-values that are significant at *p* < 0.05.

Given the non-significant results in time-lag variables from the first model, time-lags were not considered in the remaining two modelling approaches.

#### Modelling approach 2: Impact of principal components of meteorological variables on seroconversion

**Figure 4.**
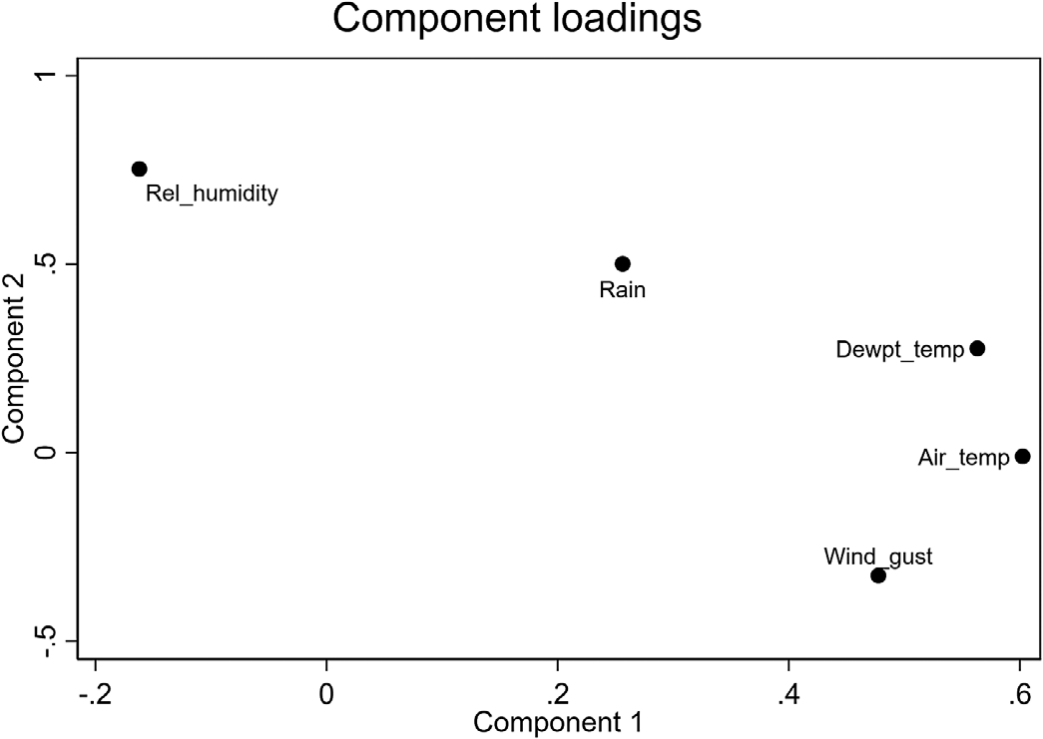
Component loadings derived from a principal component analysis of meteorological covariates collected from The University of Queensland Gatton campus weather station (station no. 040082) between 1 August 2020 and 3 July 2023. Rel_humidity = Relative humidity; Dewpt_temp = Dew point temperature; Air_temp = air temperature.

Principal component analysis identified five components, with three components having Eigenvalues <1, therefore being excluded from further analysis (Supplementary Table S2). Temperature related variables and wind gust had high positive loadings in component one, while moisture related variables had high positive loadings in component two (Figure 4). In the univariate analysis, an overall increase in each unit of component one (temperature-driven) led to an increased hazard ratio of seroconversion by 2.3 times (*p* = 0.013; Table 3). Component two (moisture-driven) was not significantly associated with seroconversion (*p* = 0.086; Table 3). Results of the goodness-of-fit test are shown in Supplementary Figure S15.

**Table 3.** Univariate Cox regression results of the association between time till Ross River virus seroconversion and principal components of meteorological variables (modelling approach 2)

Component	Hazard ratio (95% CI)	*p* value
1	2.30 (1.20, 4.43)	**0.013**
2	0.60 (0.33, 1.08)	0.086

Bold entries highlight *p*-values that are significant at *p* < 0.05.

#### Modelling approach 3: Impact of percent change of individual meteorological variables compared to previous month on seroconversion

The univariate analysis in the third modelling approach revealed that every percent change in air temperature, dew point temperature, and wind gust compared to the previous month resulted in significantly increased hazard ratios by 2.9, 1.4, and 2.0 times, respectively (Table 4). The final multivariate model only included air temperature and wind gust, with both increasing significantly (*p* < 0.05) the hazard ratio for seroconversion (Table 5). Results of the goodness-of-fit assessment are shown in Supplementary Figure S16.

**Table 4.** Univariate Cox regression results of the association between time till Ross River virus seroconversion and standardized meteorological covariates expressed in percent change (modelling approach 3)

Variable	HR (95% CI)	*p* value
Air temperature (°C)	2.89 (1.33, 6.29)	**0.007**
Dew point temperature (°C)	1.39 (1.01, 1.93)	**0.046**
Relative humidity (%)	0.51 (0.18, 1.43)	0.204
Wind gust (knots)	2.03 (1.21, 3.42)	**0.007**
Cumulative Rainfall (mm)	1.00 (0.96, 1.04)	0.903

Bold entries highlight *p*-values that are significant at *p* < 0.05.

**Table 5.** Multivariate Cox regression results of the association between time till Ross River virus seroconversion and standardized meteorological covariates expressed in percent change (modelling approach 3)

Variable	HR (95% CI)	*p* value
Air temperature (°C)	3.11 (1.24, 7.79)	**0.015**
Wind gust (knots)	1.96 (1.14. 3.39)	**0.016**

Bold entries highlight *p*-values that are significant at *p* < 0.05.

## Discussion

Horses constitute one of the very few domestic mammalian species that are naturally susceptible to many arbovirus infections of medical concern and they display clinical signs similar to those of infected humans. In Australia, RRV infection is a notifiable disease in humans. The last annual report (2014–2015) on human infection was published in 2019 indicating that RRV remains the most prevalent arbovirus infection [34]. Since RRV infection in animals is non-notifiable in Australia, the prevalence of arbovirus infection is only known from published serological surveys. Since an outbreak of neurological arboviral disease in horses in 2011, where RRV infection was also reported in South Australia [35], there have only been three serological surveys conducted in horses [16, 36, 37]. Currently an active Australian National Arbovirus Monitoring Programme is conducted in cattle, sheep, and goats to monitor the transmission of bluetongue virus, Akabane virus, and bovine ephemeral fever virus [38]. These viruses are primarily transmitted by *Culicoides* spp. and can have a significant impact on livestock production, but they do not infect humans and horses and hence are not considered of public health concern.

Maternal antibodies protect foals from infection for the first three to four months of life post-partum. After this time, maternal neutralizing antibody levels fall below the detectable limit but may still interfere with endogenous immunoglobulin production in the foal upon infection or vaccination until six months of age [39, 40]. In the present study, protective levels of maternal antibodies specific for RRV in foals born to RRV seropositive mares were detectable for 4.6 months, with a 95% confidence interval of 3.8 to 5.1 months. Once maternal antibodies declined to non-protective levels (< 1:20 in VNT), foals are susceptible to natural infection of RRV by the bite of an infected mosquito. Interestingly, the foals in the current study were at risk of infection for 14.3 months (with a 95% confidence interval of 9.8 to 19.4 months) and all seroconversion events occurred between December and April consistently across all three foal cohorts. This supports the suggestion that naïve horses may be suitable sentinel animals for RRV circulation considering that notification rates in humans in SE QLD consistently peak later (between March and May), as identified by several 10-year retrospective studies [41, 42].

Across all three modelling approaches, temperature-related variables, especially air temperature, were consistently associated with seroconversion. As seroconversion in horses occurs over summer to early autumn (December to March), it is not surprising that the increase in temperature increased the hazard ratio for seroconversion. It appears that seroconversion events occurred when the averaged air temperature was approximately between 18 °C and 25 °C. This is likely related to mosquito activities as larvae development and host-seeking activities are optimal between 15 °C and 32 °C [43, 44], and as mosquito activity ceases when temperature falls below the minimum metabolic requirements [45]. In addition, percent change in wind gust was significantly associated with seroconversion. While the hazard ratio for percent change in model approach 3 does not indicate whether a positive or negative percent change in wind gust increases the hazard ratio, it is likely that the decrease in wind gust increases the hazard ratio as strong wind would disperse mosquitoes [46] and decrease air temperature, thereby reducing overall mosquito activities and thus limiting the chances for infection to occur.

Owing to the differences in landscape and climate features across Australia, the transmission of RRV varies across different regions even within a state. For example, transmission of RRV occurs year-round in northern QLD [21, 41, 47], but it is only seasonal in SE QLD [25]. Studies identified a seroprevalence of RRV in horses at 91% in northern QLD [37], and approximately at 50% in SE QLD, with the location for the current study having the highest prevalence at 85% [16]. Results from the present study confirmed that transmission of RRV in horses in SE QLD follows a seasonal pattern with seroconversion events only being detected between December and March consistently throughout a 3.5-year period and being associated with air temperature without time-lag. In contrast, epidemiological modelling using human notification data in QLD showed that human infection peaked between March and May with rainfall, relative humidity, and air temperature identified as risk factors with 0–3 months’ time-lags [5, 17–24]. Year-round irrigation usage and filled water troughs on farms and in paddocks in this subtropical study area would provide ideal breeding grounds for mosquitoes. Within urban areas, the presence of water puddles after rainfall, poorly maintained swimming pools, bird baths, and water accumulating in plant pots may provide more short-term breeding grounds for mosquitoes. Also, in regional and rural areas, reservoir hosts, such as marsupials, may be present in larger numbers than in build-up areas and would maintain the transmission cycle all year. With the close proximity of mosquito breeding grounds and the shared habitat between reservoir hosts and horses, it is not surprising that a time-lag effect was not observed in this study.

Overall, this further supports the conjecture that RRV infection in horses may provide a proxy for increased RRV transmission in the human population. Real time monitoring of seroconversion events in horses in peri-urban areas may be more effective and reliable than predictions from mathematical modelling which uses historical data. This is especially true in the era of climate change as meteorological factors change unpredictably, making disease transmission forecasting through mathematical models very challenging. While mosquito surveillance programmes provide information about arboviruses circulating in the mosquito population, they do not confirm active exposure dynamics between vector and susceptible hosts. A near real-time monitoring of active circulation of RRV in horses would support the implementation of time-sensitive public health measures. Moreover, the seroprevalence of 50% in horses residing in southern QLD means that the transmission of RRV in the horse population is high enough to provide confidence in detecting seroconversion events in any given year [16].

There are a few considerations before conducting such real-time monitoring. Firstly, a young horse population is preferable, as horses older than 6 years of age are two times more likely to have already seroconverted than 2–6 years old [16]. Secondly, strategic or risk-based sampling would lower the costs of these studies. Given transmission in SE QLD consistently starts in December, sampling could begin in mid-spring each year and end in autumn when human cases would have peaked. Thirdly, a simple ELISA test or point-of-care test (e.g., lateral flow assay) could be developed and used as a diagnostic tool. As the goal of such monitoring is to detect the presence of neutralizing antibodies, the labour-intensive and time-consuming gold standard virus neutralization assay is not required to determine antibody titre. This would increase the turn-around time and help lower the cost of laboratory testing. In addition, since Getah virus remains exotic to Australia [48] and since no alphavirus vaccine is currently available in Australia, antibody cross-neutralization would be of minimal concern. A similar monitoring approach could be adapted across Australia, and in neighbouring countries where RRV is endemic, or spillover events have been sporadically detected.

The study presented here used the presence of neutralizing antibodies as an indicator of infection. However, detectable seroconversion only occurs approximately two weeks after infection. Therefore, it should be acknowledged that a two-week lag exists in the present data. However, since blood sample collection was conducted on a monthly basis and analyses of meteorological risk factors were summarized monthly, the two-week lag effect of the detection of seroconversion from initial infection would have little-to-no impact on the analysis results. While mosquito testing was not performed to confirm the presence of RRV in the vector, mosquito trappings revealed that the majority of the mosquito population on the UQ Gatton campus consists of *Culex annulirostris* (data not shown), a known competent, efficient, and important vector for RRV transmission [6].

## Conclusion

This study represents the first prospective longitudinal study of RRV transmission in a region of Australia with high transmission rate. In summary, the increase in air temperature and probably decrease in wind gust are associated with seroconversion events in horses without time-lag effect. These results support the use of horses as sentinel animals for RRV transmission to inform public health measures and horses could potentially be integrated into an early warning detection system of RRV epidemics or outbreaks. Given the importance of RRV infection medically and economically in Australia, serosurveillance of horses in peri-urban areas as in parallel with real-time human notification data in surrounding regions (a One Health approach) is warranted to confirm the suitability and effectiveness of using horses as sentinels for RRV infection.

## Supporting information

Yuen et al. supplementary material Yuen et al. supplementary material

## Data Availability

All data have been presented in the manuscript and Supplementary Material.

## Supplementary material

For supplementary material accompanying this paper visit http://doi.org/10.1017/S0950268824000554. click here to view supplementary material
