# Supplementary material for: Exposure dynamics of Ross River virus in horses – Horses as potential sentinels (a One Health approach)
Source: Epidemiol Infect. 2024 Apr 12;152:e67. doi: 10.1017/S0950268824000554 (PMC11062785; doi:10.1017/S0950268824000554)
Supplement: Yuen et al. supplementary material [file S0950268824000554sup001.docx]

*Epidemiology and Infection*

Exposure dynamics of Ross River virus in horses – horses as potential sentinels (a One Health approach)

Nicholas K. Y. Yuen, Helle Bielefeldt-Ohmann, Mitchell P. Coyle, Joerg Henning

Supplementary material

**Supp Table S1.** Summary statistics of survival time for the waning of Ross River virus neutralising maternal antibodies and Ross River virus seroconversion in 32 horses monitored over 3.5 years in South East Queensland, Australia.

| **Category** | **Year** | **Survival time (days)** | | |
| --- | --- | --- | --- | --- |
|  |  | **Median** | **95% CI (low)** | **95% CI (high)** |
| Maternal antibodies | Overall | 137 | 113 | 154 |
|  | 2020 | 188 | 109 | 208 |
|  | 2021 | 138 | 58 | 168 |
|  | 2022 | 114 | 33 | 127 |
| Seroconversion | Overall | 429 | 294 | 582 |
|  | 2020 | 364 | 133 | 481 |
|  | 2021 | 454 | 255 | N/A |
|  | 2022 | N/A | 128 | N/A |

N/A = Theses values could not be calculated through non-parametric Kaplan-Meier survival estimation.


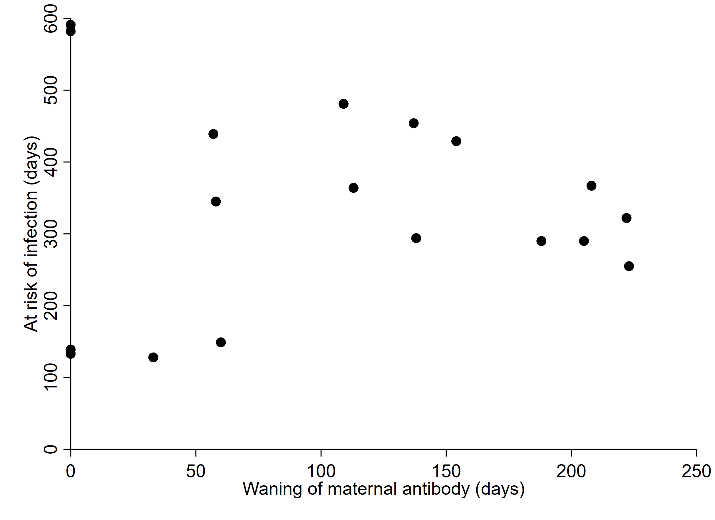


**Supp Figure S1.** Scatterplot of time (in days) for waning of maternal Ross River virus specific neutralising antibodies and Ross River virus seroconversion (in days) in the same horses monitored over 3.5 years in South East Queensland, Australia.


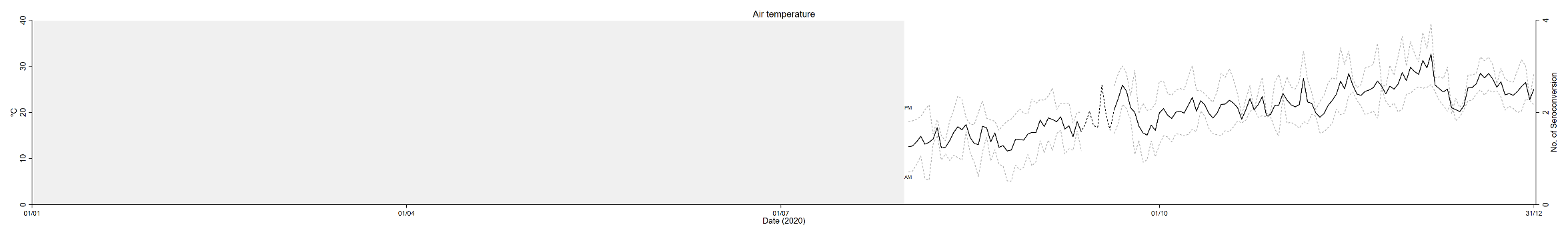

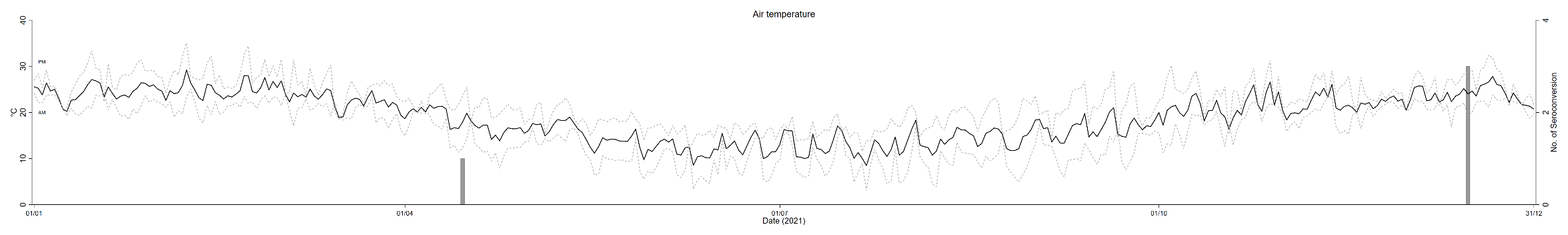

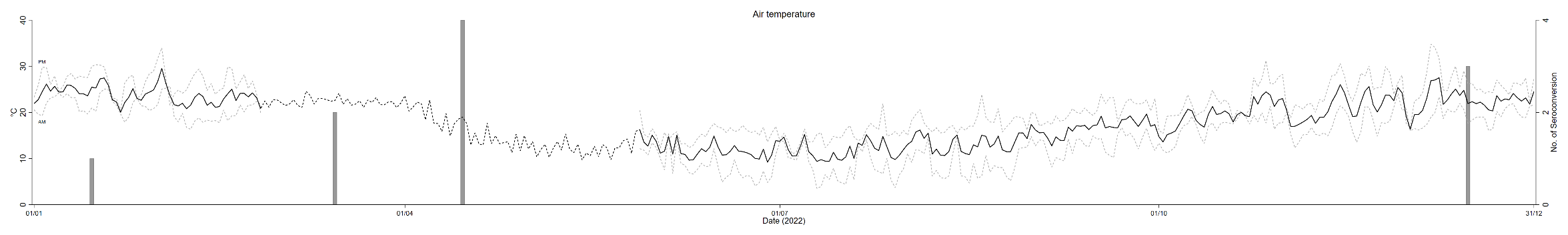

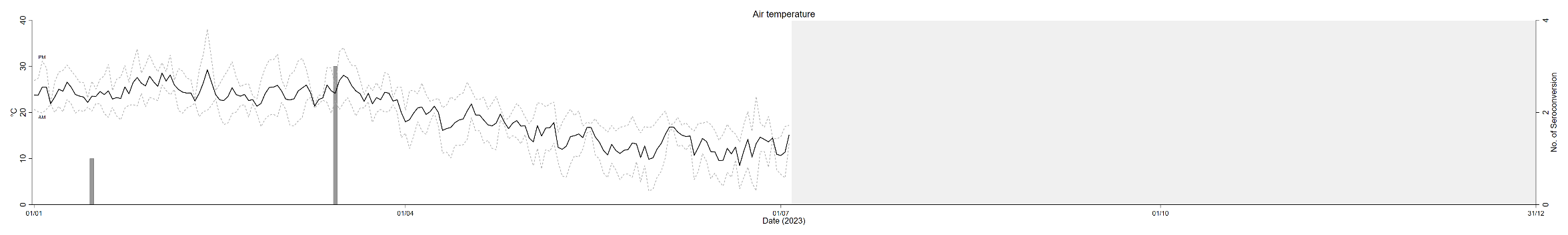


**Supp Figure S2.** Averaged daily air temperature from August 2020 to July 2023 (black solid line) in longitudinal study on natural Ross River virus infection in horses in South East Queensland, Australia. Data were collected at the University of Queensland Gatton Campus weather station (station no. 040082). The dotted line in black refers to imputed data (see section 2.6). The dotted line in grey refers to averaged AM (0500 – 0800) or PM (1600 – 1900) temperature. Grey bars indicate the number of horses naturally infected with Ross River virus at the relevant time point. Area shaded in grey indicates time of the year before commencement or after conclusion of the study period.


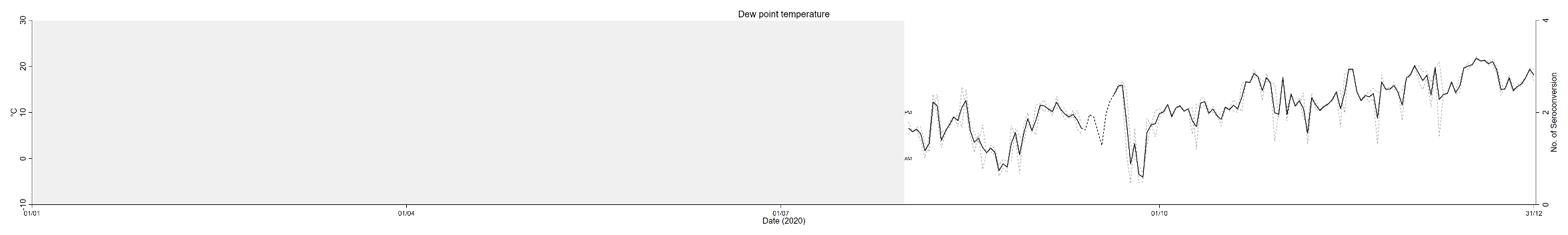

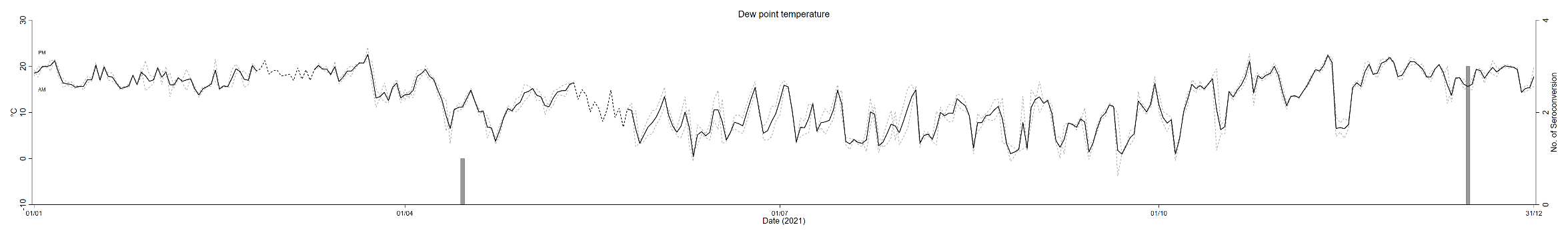

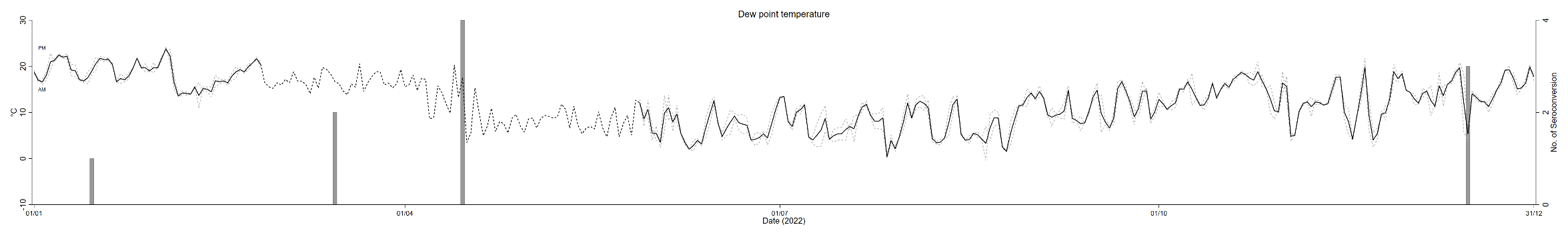

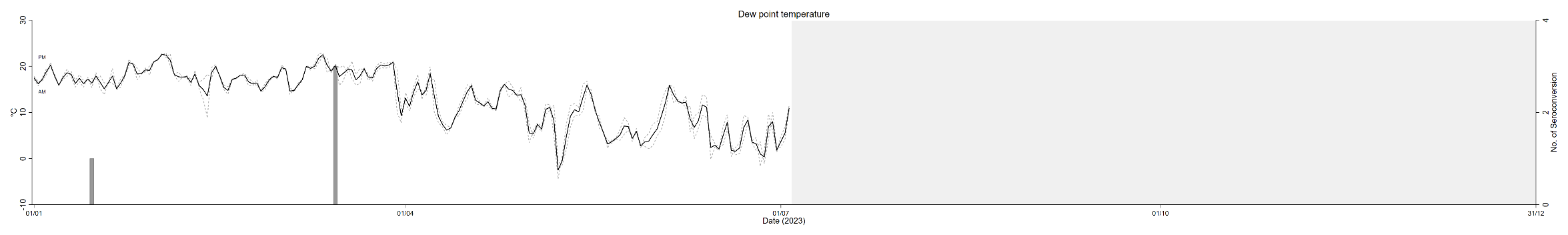


**Supp Figure S3.** Averaged daily dew point temperature from August 2020 to July 2023 (black solid line) in longitudinal study on natural Ross River virus infection in horses in South East Queensland, Australia. Data were collected at the University of Queensland Gatton Campus weather station (station no. 040082). The dotted line in black refers to imputed data (see section 2.6). The dotted line in grey refers to averaged AM (0500 – 0800) or PM (1600 – 1900) temperature. Grey bars indicate the number of horses naturally infected with Ross River virus at the relevant time point. Area shaded in grey indicates time of the year before commencement or after conclusion of the study period.


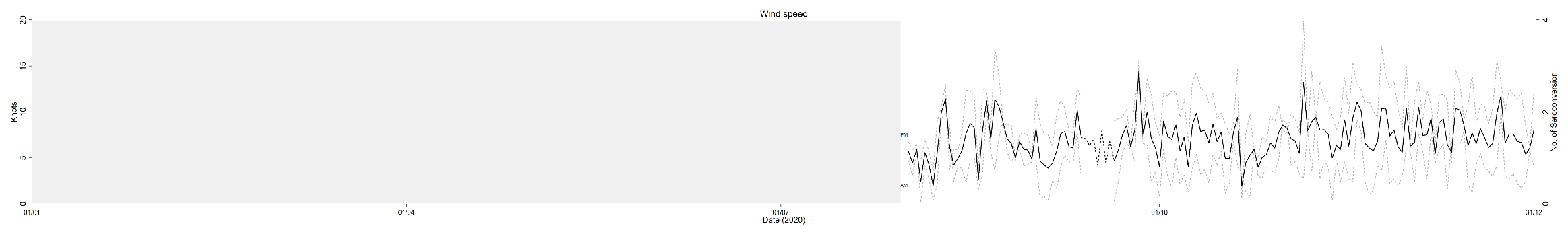

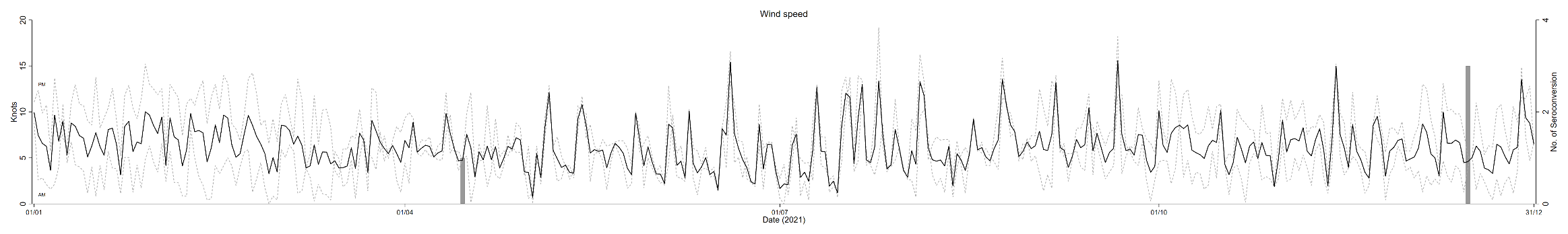

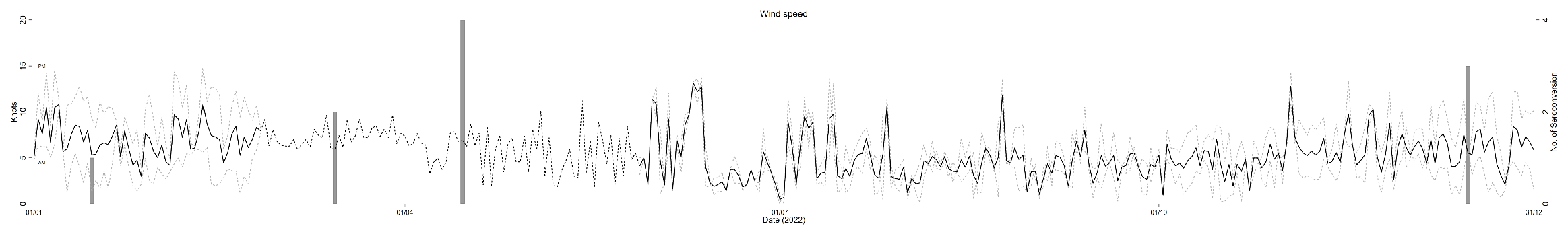

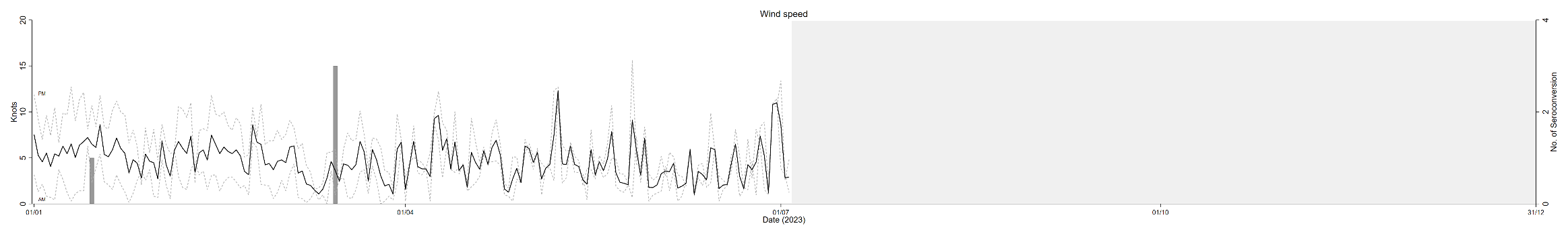


**Supp Figure S4.** Averaged daily wind speed from August 2020 to July 2023 (black solid line) in longitudinal study on natural Ross River virus infection in horses in South East Queensland, Australia. Data were collected at the University of Queensland Gatton Campus weather station (station no. 040082). The dotted line in black refers to imputed data (see section 2.6). The dotted line in grey refers to averaged AM (0500 – 0800) or PM (1600 – 1900) temperature. Grey bars indicate the number of horses naturally infected with Ross River virus at the relevant time point. Area shaded in grey indicates time of the year before commencement or after conclusion of the study period.


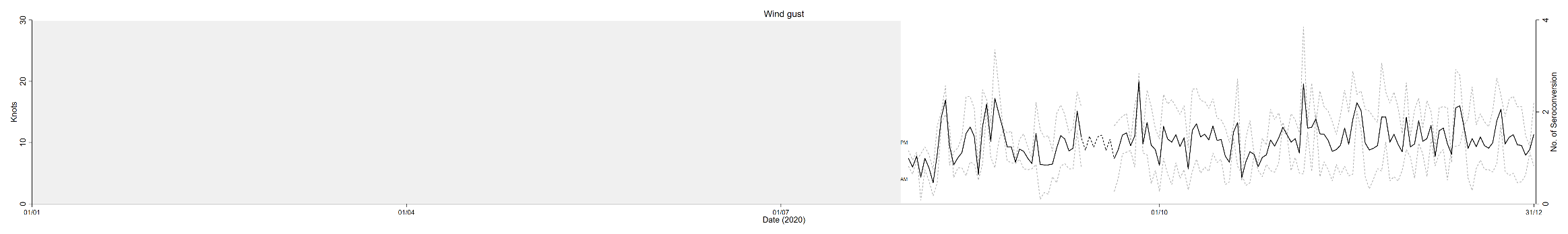

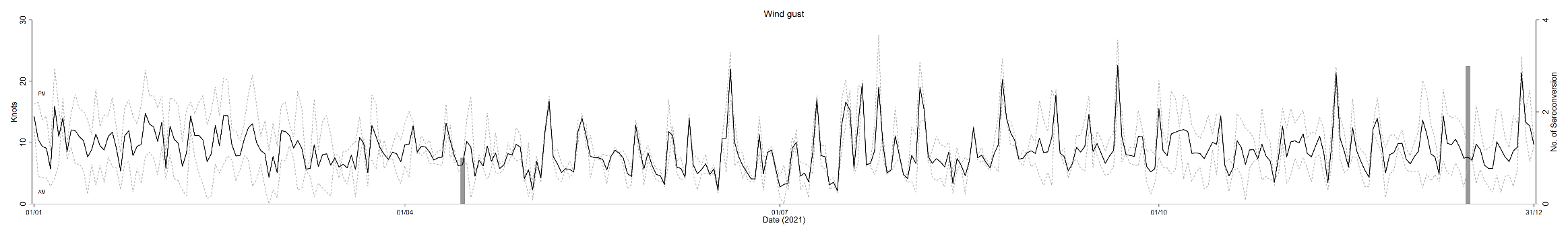

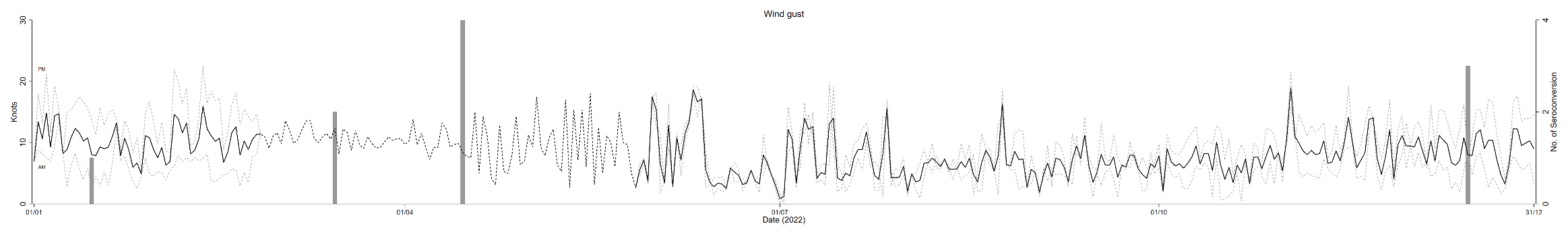

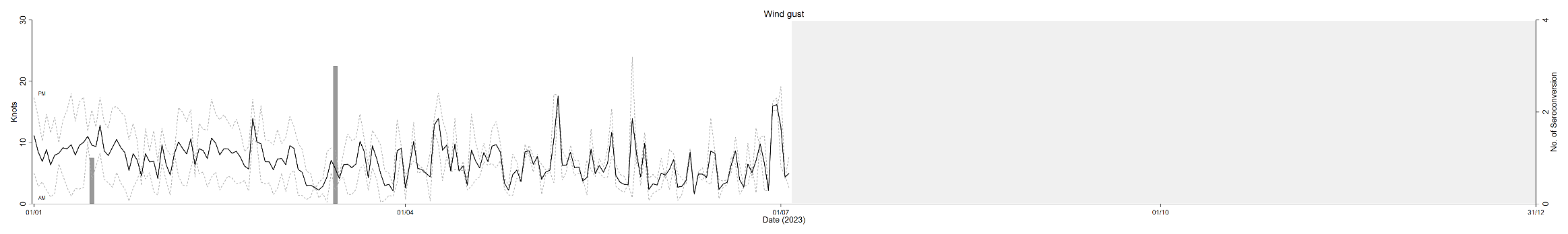


**Supp Figure S5.** Averaged daily wind gust from August 2020 to July 2023 (black solid line) in longitudinal study on natural Ross River virus infection in horses in South East Queensland, Australia. Data were collected at the University of Queensland Gatton Campus weather station (station no. 040082). The dotted line in black refers to imputed data (see section 2.6). The dotted line in grey refers to averaged AM (0500 – 0800) or PM (1600 – 1900) temperature. Grey bars indicate the number of horses naturally infected with Ross River virus at the relevant time point. Area shaded in grey indicates time of the year before commencement or after conclusion of the study period.


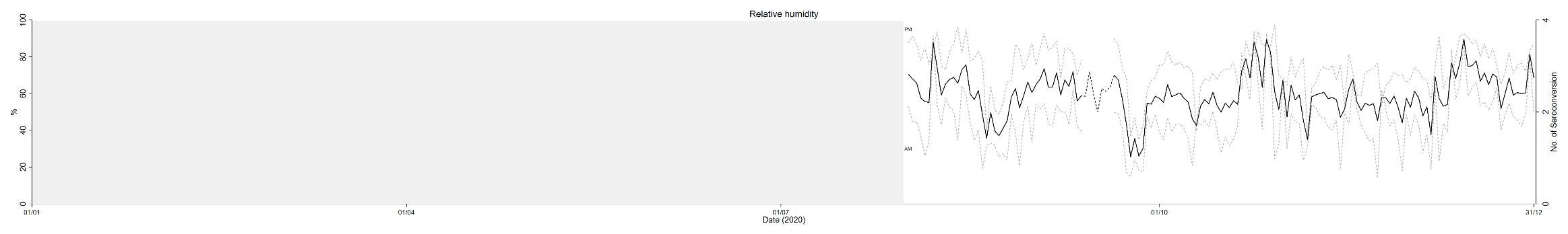

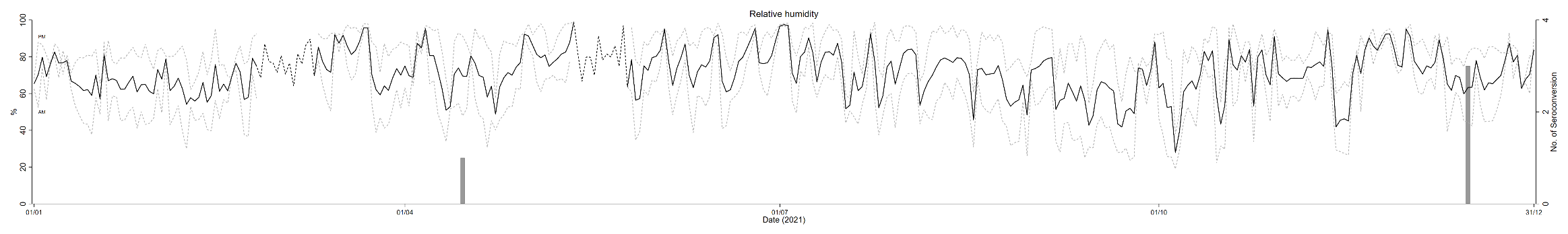

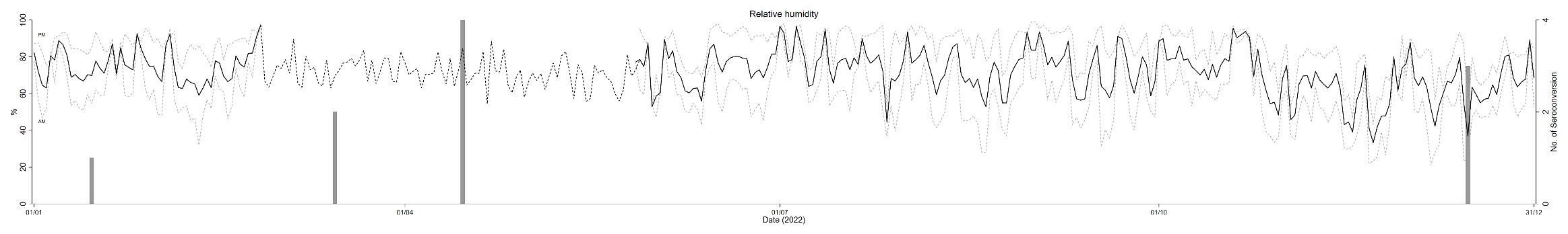

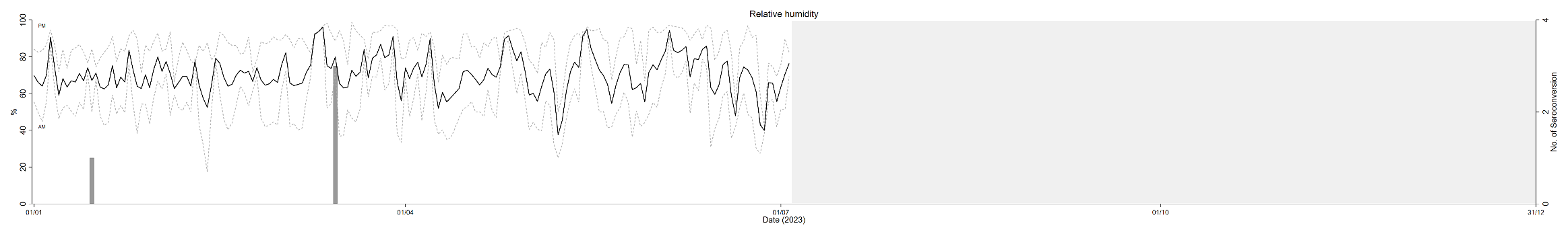


**Supp Figure S6.** Averaged daily relative humidity from August 2020 to July 2023 (black solid line) in longitudinal study on natural Ross River virus infection in horses in South East Queensland, Australia. Data were collected at the University of Queensland Gatton Campus weather station (station no. 040082). The dotted line in black refers to imputed data (see section 2.6). The dotted line in grey refers to averaged AM (0500 – 0800) or PM (1600 – 1900) temperature. Grey bars indicate the number of horses naturally infected with Ross River virus at the relevant time point. Area shaded in grey indicates time of the year before commencement or after conclusion of the study period.


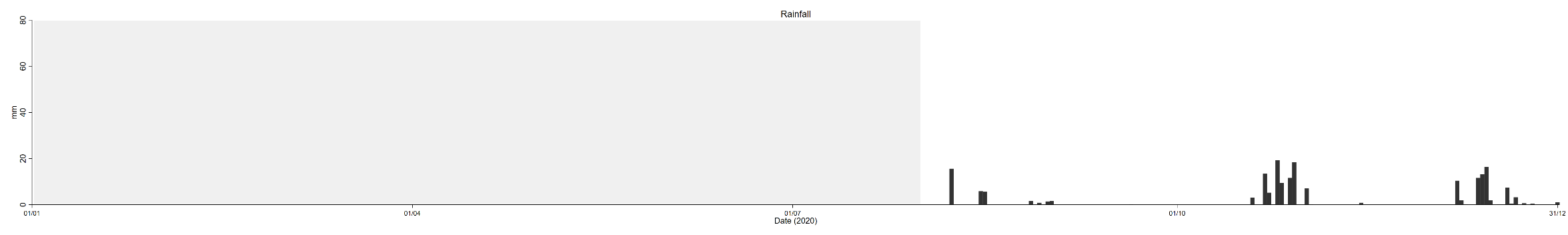

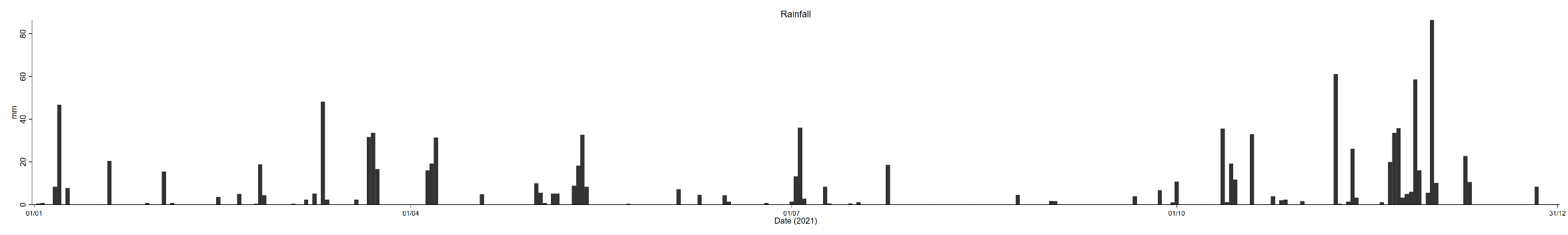

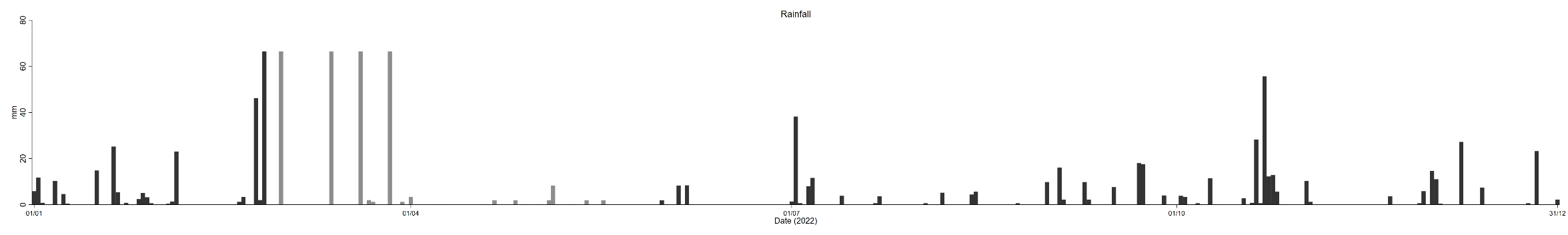

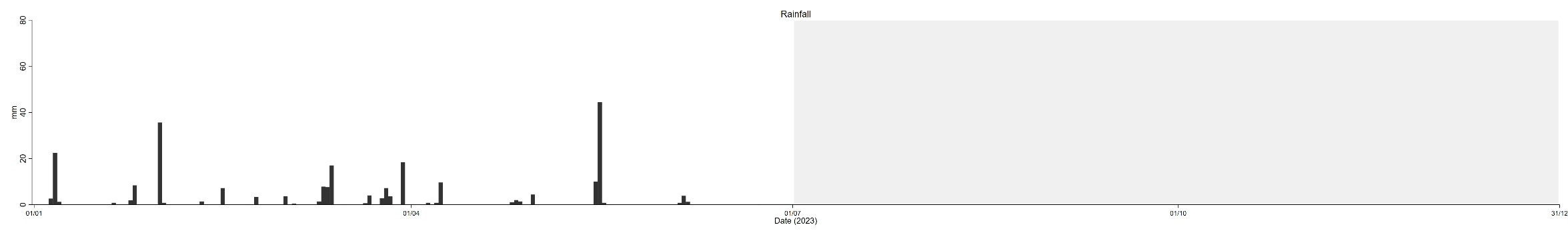


**Supp Figure S7.** Cumulative daily rainfall from August 2020 to July 2023 (black) in longitudinal study on natural Ross River virus infection in horses in South East Queensland, Australia. Data were collected at the University of Queensland Gatton Campus weather station (station no. 040082). The bars in grey refers to imputed data (see section 2.6). Area shaded in grey indicates time of the year before commencement or after conclusion of the study period.


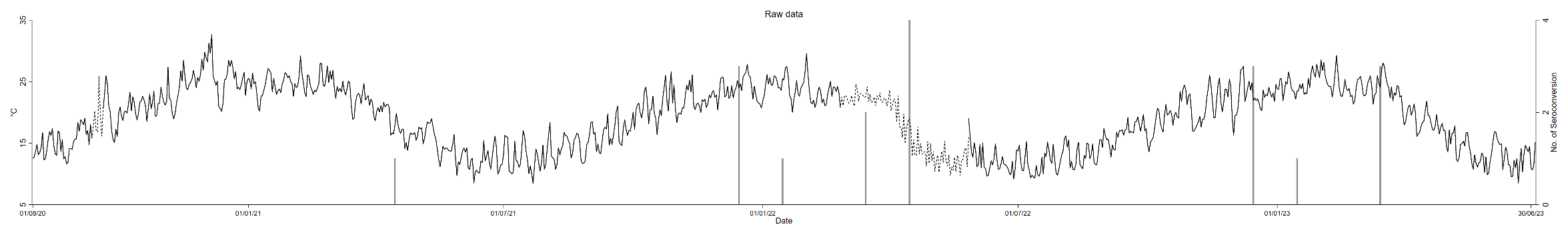

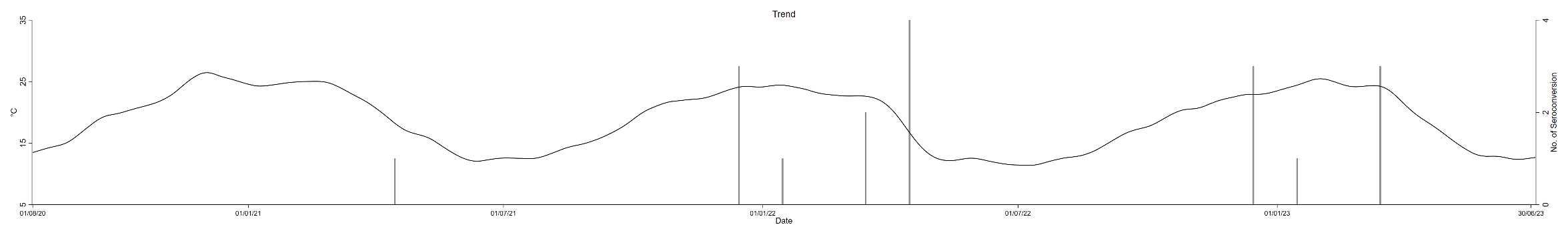

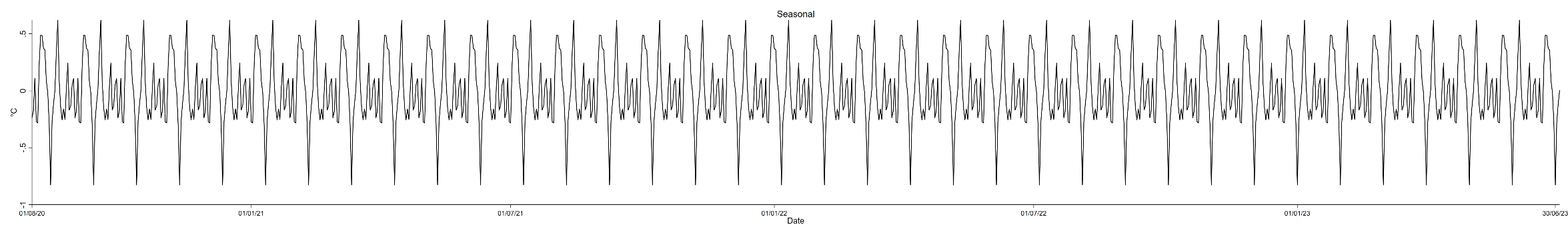

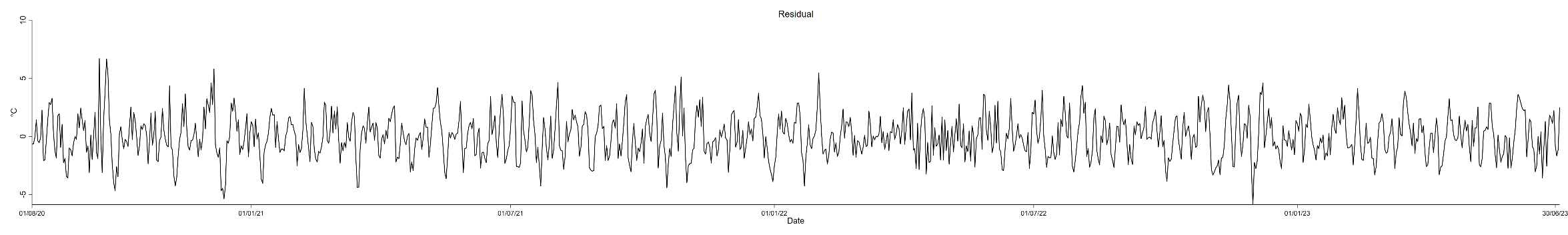


**Supp Figure S8.** Time series decomposition of daily averaged air temperature in longitudinal study on natural Ross River virus infection in horses in South East Queensland, Australia. Data were collected at the University of Queensland Gatton Campus weather station (station no. 040082). The dotted line in black refers to imputed data (see section 2.6). Raw data = Trend + Seasonal + Residual. Grey bars indicate the number of horses naturally infected with Ross River virus at the relevant time point.


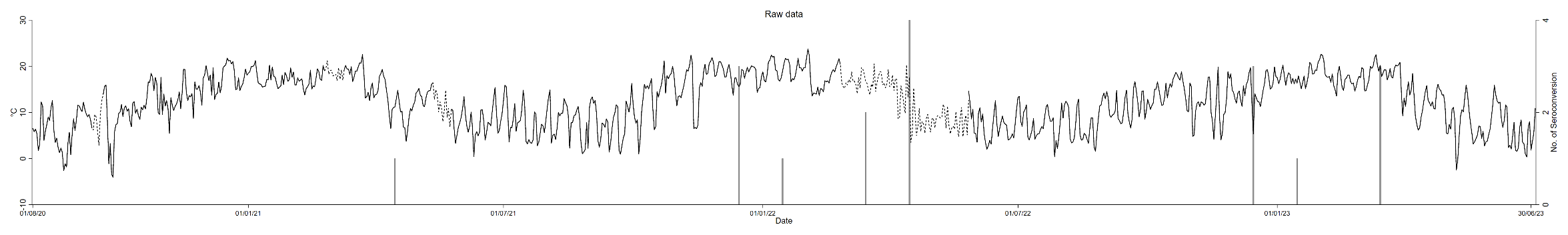

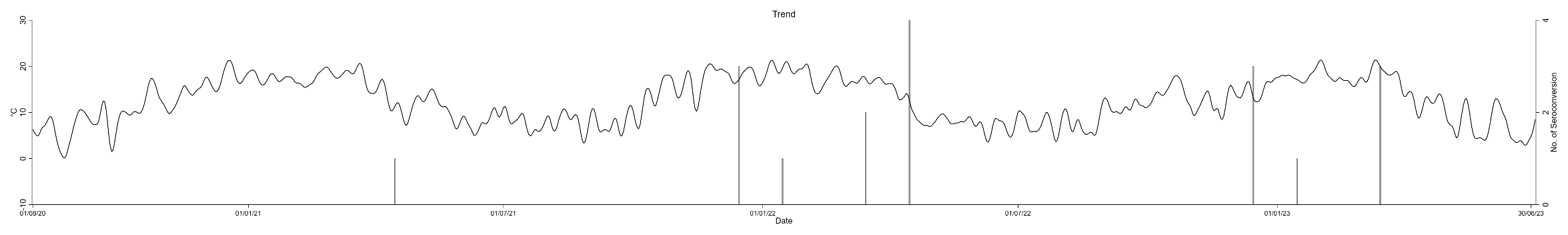

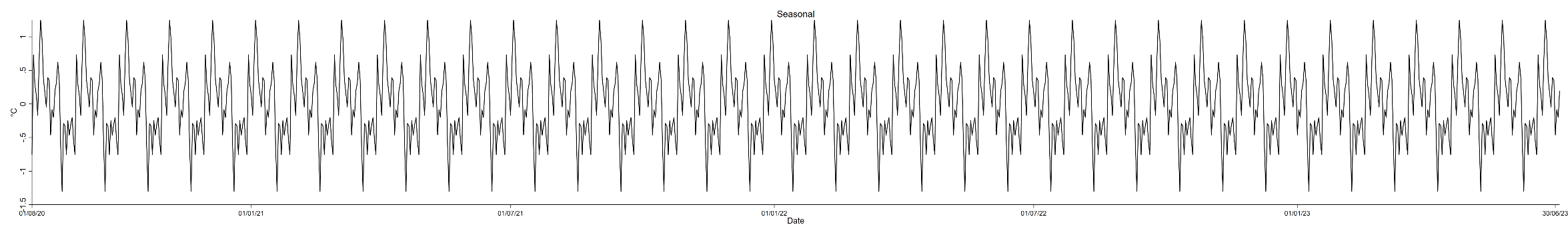

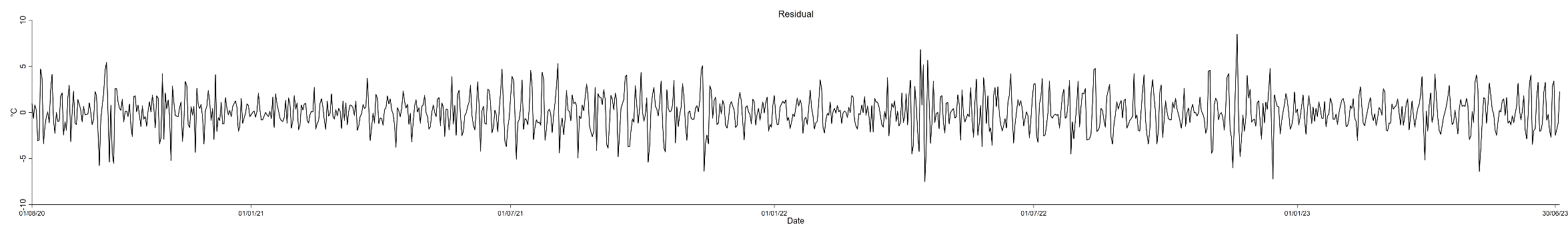


**Supp Figure S9.** Time series decomposition of daily averaged dew point temperature in longitudinal study on natural Ross River virus infection in horses in South East Queensland, Australia. Data were collected at the University of Queensland Gatton Campus weather station (station no. 040082). The dotted line in black refers to imputed data (see section 2.6). Raw data = Trend + Seasonal + Residual. Grey bars indicate the number of horses naturally infected with Ross River virus at the relevant time point.


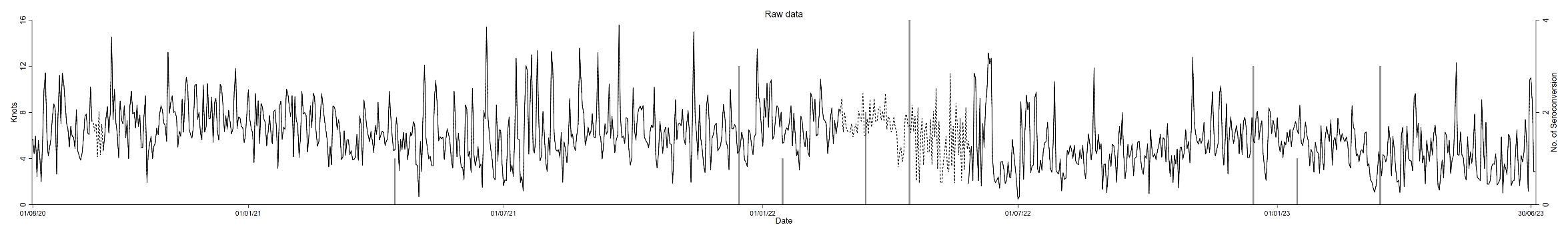

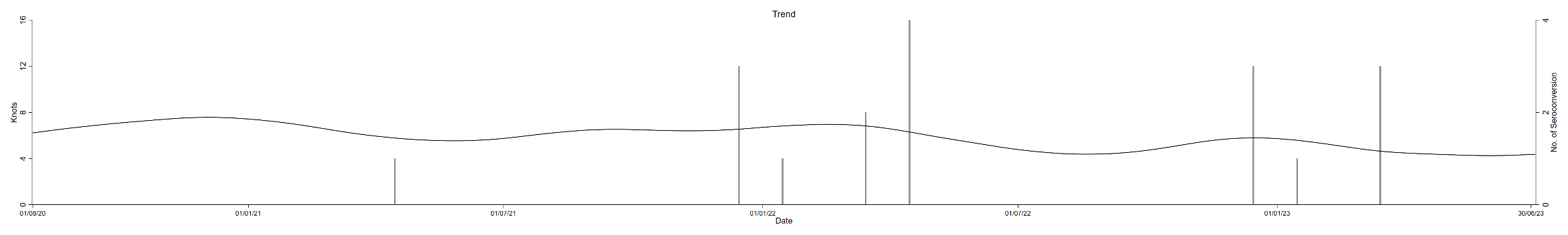

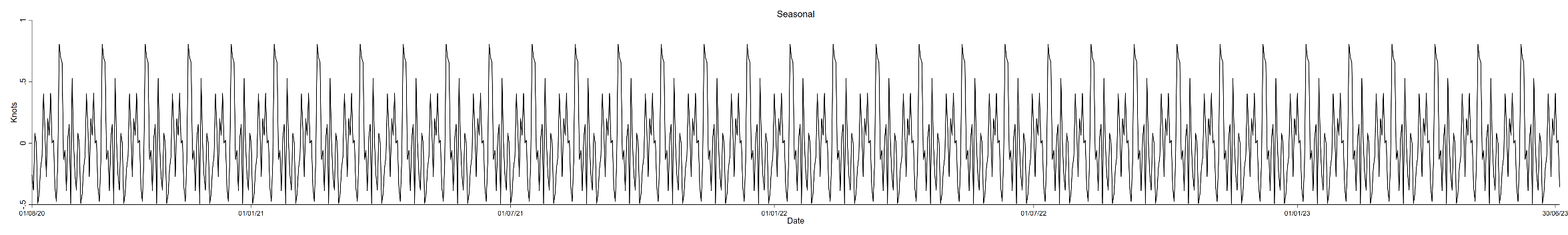

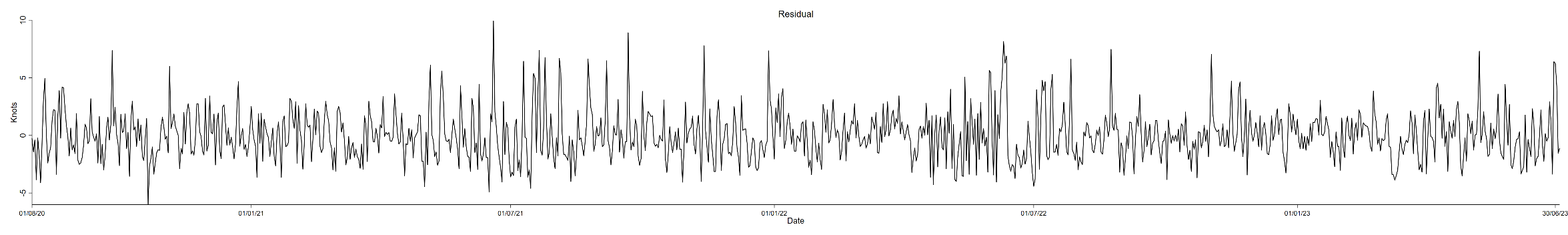


**Supp Figure S10.** Time series decomposition of daily averaged wind speed in longitudinal study on natural Ross River virus infection in horses in South East Queensland, Australia. Data were collected at the University of Queensland Gatton Campus weather station (station no. 040082). The dotted line in black refers to imputed data (see section 2.6). Raw data = Trend + Seasonal + Residual. Grey bars indicate the number of horses naturally infected with Ross River virus at the relevant time point.


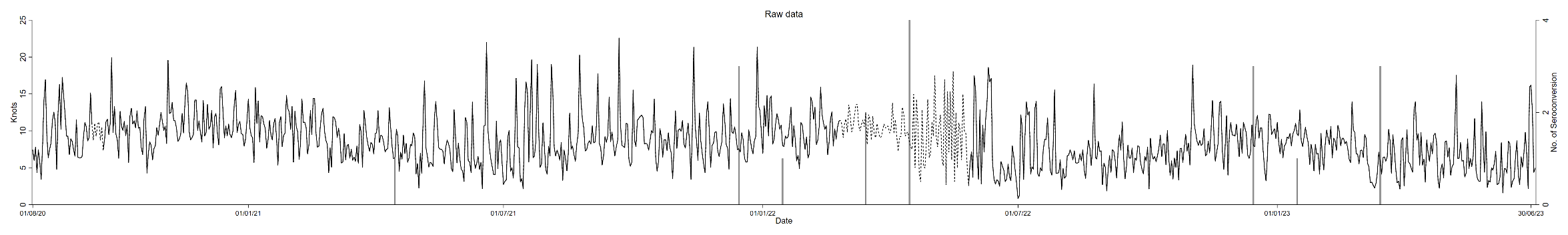

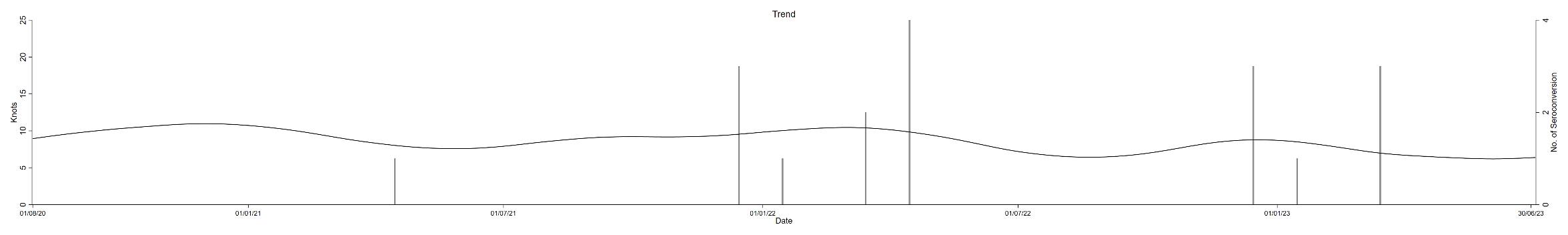

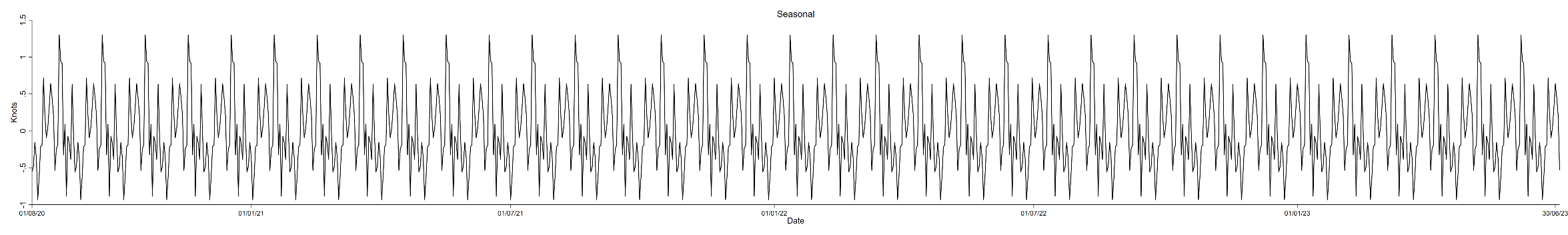

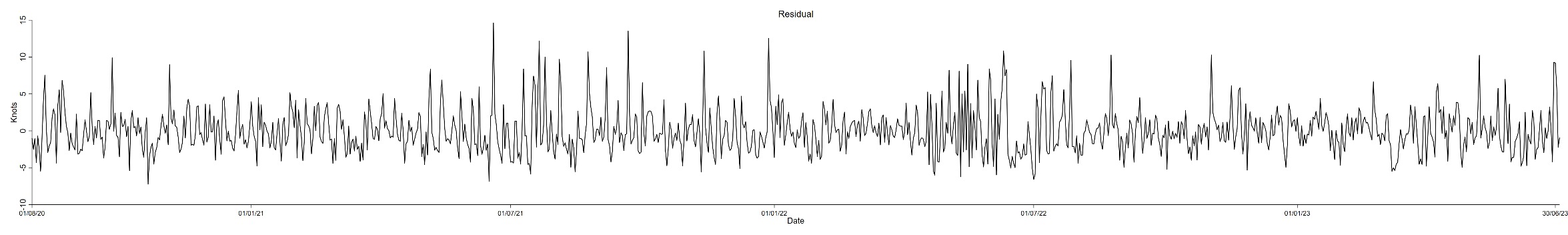


**Supp Figure S11.** Time series decomposition of daily averaged wind gust in longitudinal study on natural Ross River virus infection in horses in South East Queensland, Australia. Data were collected at the University of Queensland Gatton Campus weather station (station no. 040082). The dotted line in black refers to imputed data (see section 2.6). Raw data = Trend + Seasonal + Residual. Grey bars indicate the number of horses naturally infected with Ross River virus at the relevant time point.


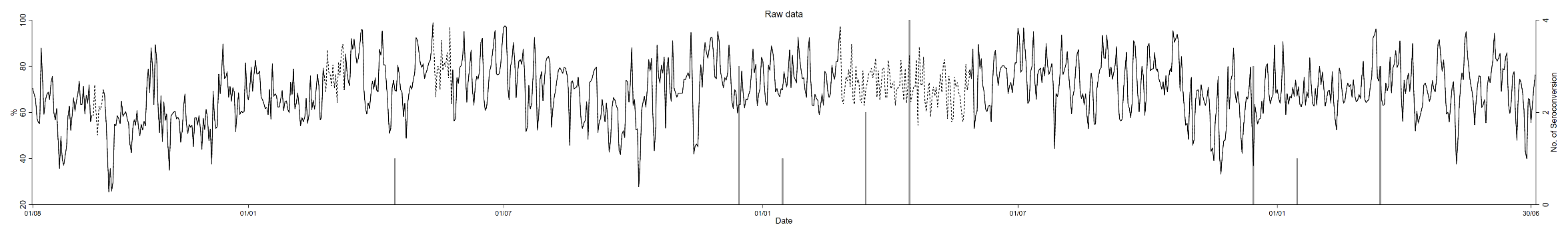

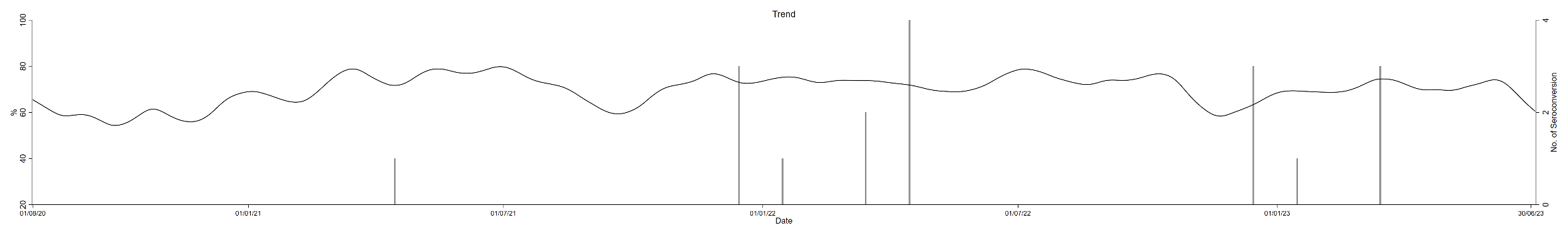

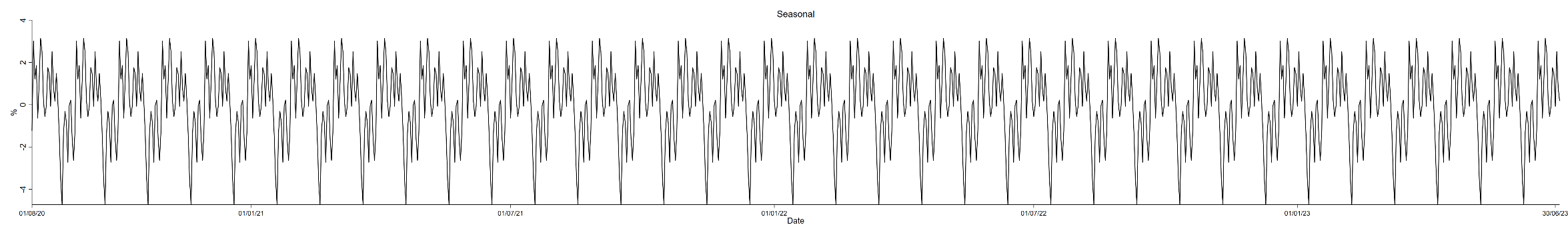

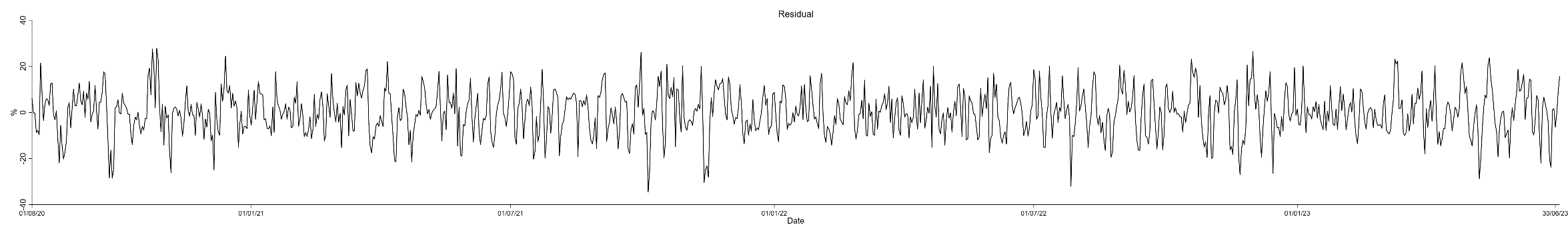


**Supp Figure S12.** Time series decomposition of daily averaged relative humidity in longitudinal study on natural Ross River virus infection in horses in South East Queensland, Australia. Data were collected at the University of Queensland Gatton Campus weather station (station no. 040082). The dotted line in black refers to imputed data (see section 2.6). Raw data = Trend + Seasonal + Residual. Grey bars indicate the number of horses naturally infected with Ross River virus at the relevant time point.


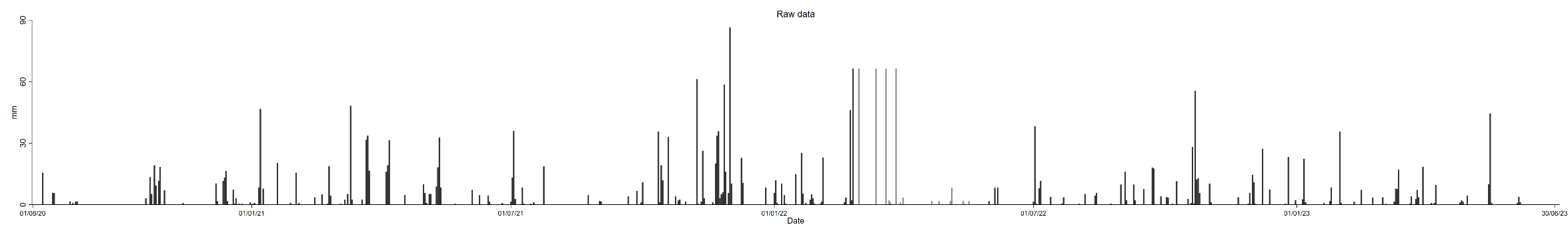

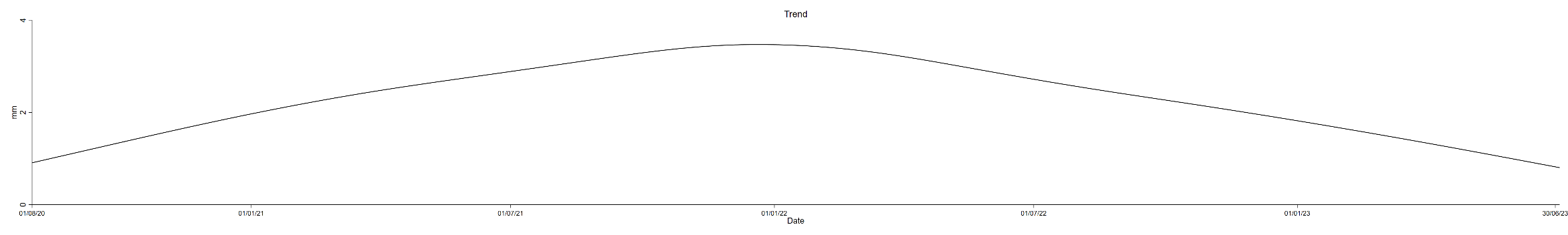

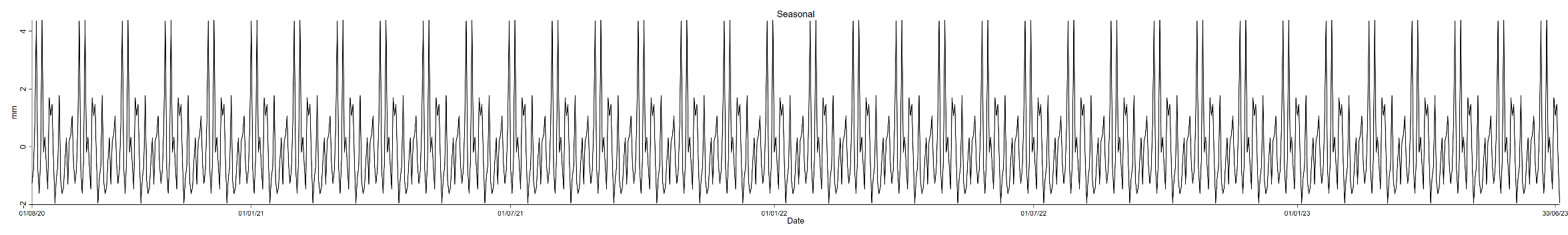

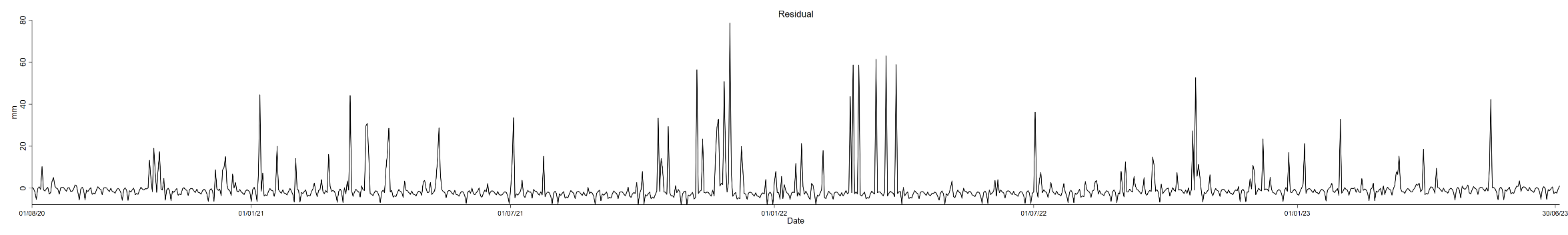


**Supp Figure S13.** Time series decomposition of daily cumulative rainfall in longitudinal study on natural Ross River virus infection in horses in South East Queensland, Australia. Data were collected at the University of Queensland Gatton Campus weather station (station no. 040082). The dotted line in black refers to imputed data (see section 2.6). Raw data = Trend + Seasonal + Residual.

A
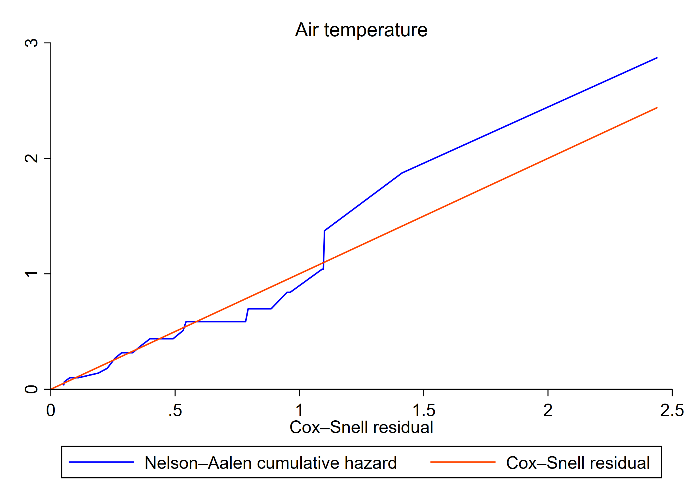
 B
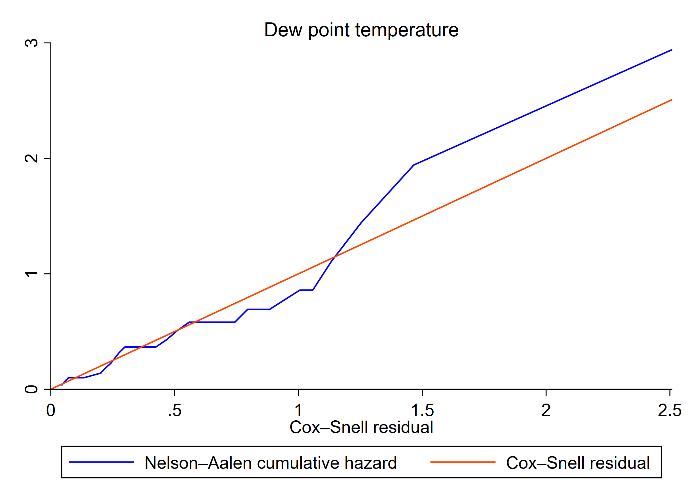
C
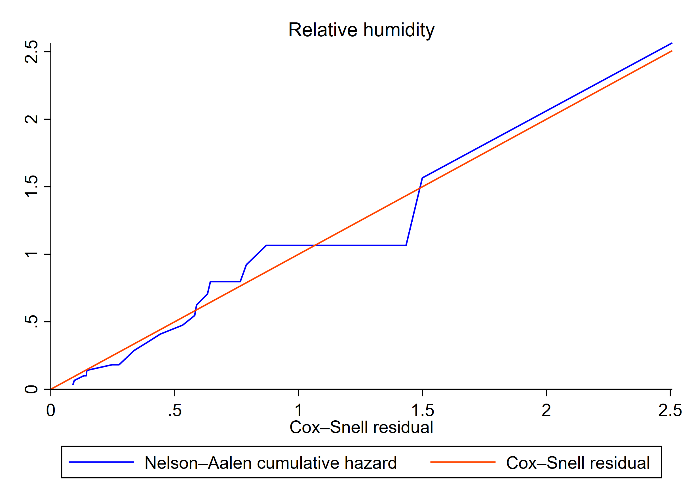
 D
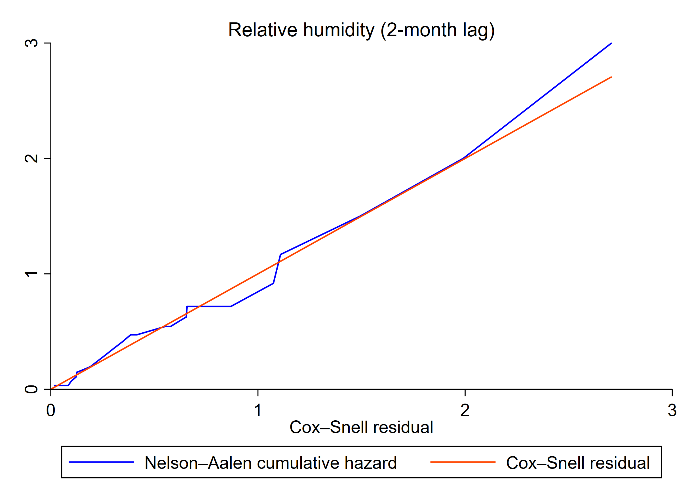


**Supp Figure S14.** Cox-Snell curve (goodness of fit assessment) for model 1; air temperature (A); dew point temperature (B); relative humidity (C); relative humidity with 2-month lag (D).

**Supp Table S2.** Results of Principal component analysis (eigenvectors scoring coefficients) for model 2.

| **Variable** | **Component 1** | **Component 2** | **Component 3** | **Component 4** | **Component 5** |
| --- | --- | --- | --- | --- | --- |
| Air temperature | 0.602 | -0.010 | -0.363 | -0.192 | 0.685 |
| Dew point temperature | 0.653 | 0.276 | -0.369 | 0.006 | -0.686 |
| Relative humidity | -0.162 | 0.752 | -0.131 | 0.574 | 0.246 |
| Wind gust | 0.478 | -0.326 | 0.472 | 0.665 | 0.012 |
| Cumulative rainfall | 0.256 | 0.501 | 0.702 | -0.437 | 0.031 |
| *Eigenvalue* | *2.417* | *1.499* | *0.861* | *0.220* | *0.003* |


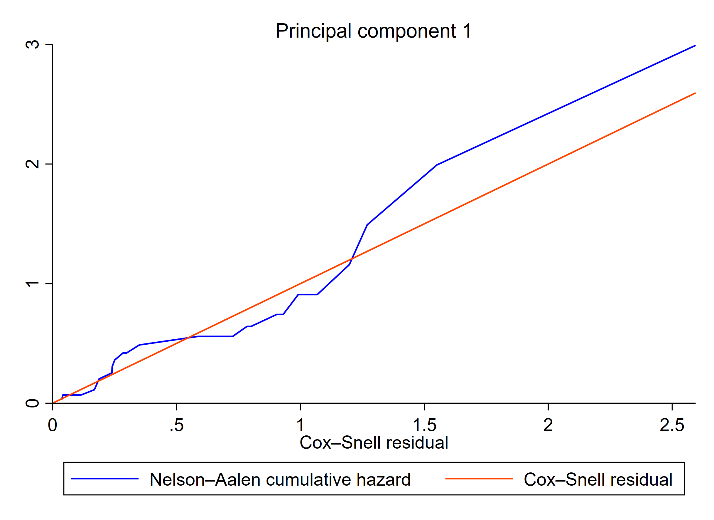


**Supp Figure S15.** Cox-Snell curve (goodness of fit assessment) for model 2.


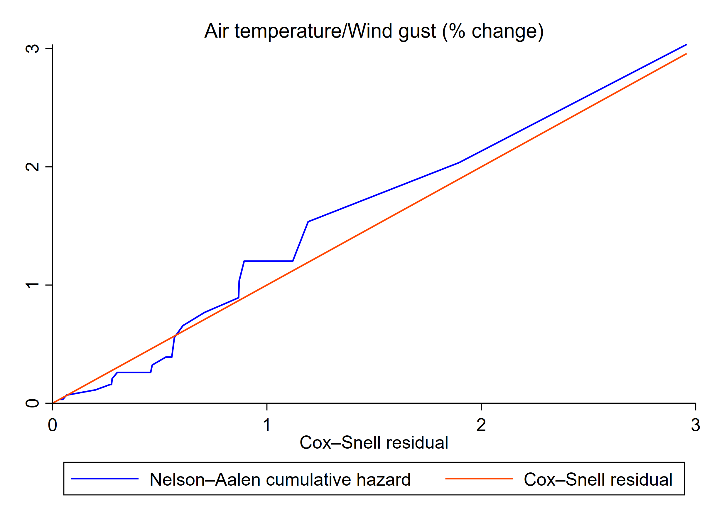


**Supp Figure S16.** Cox-Snell curve (goodness of fit assessment) for model 3.
